# Endothelial cells regulate astrocyte to neural progenitor cell trans-differentiation in a mouse model of stroke

**DOI:** 10.1038/s41467-022-35498-6

**Published:** 2022-12-19

**Authors:** Wenlu Li, Emiri T. Mandeville, Violeta Durán-Laforet, Norito Fukuda, Zhanyang Yu, Yi Zheng, Aaron Held, Ji-Hyun Park, Takafumi Nakano, Masayoshi Tanaka, Jingfei Shi, Elga Esposito, Wanting Niu, Changhong Xing, Kazuhide Hayakawa, Ignacio Lizasoain, Klaus van Leyen, Xunming Ji, Brian J. Wainger, Maria A. Moro, Eng H. Lo

**Affiliations:** 1 Neuroprotection Research Laboratories, Massachusetts General Hospital, Harvard Medical School, Boston, MA USA; 2 Unidad de Investigación Neurovascular, Departamento de Farmacología, Facultad de Medicina, Universidad Complutense de Madrid (UCM), Instituto de Investigación Hospital 12 de Octubre, Madrid, Spain; 3 Department of Neurology, Sean M. Healey & AMG Center for ALS, Massachusetts General Hospital, Harvard Medical School, Boston, MA USA; 4 Cerebrovascular Research Institute, XuanWu Hospital, Capital Medical University, Beijing, China; 5 Tissue Engineering Laboratories, VA Boston Healthcare System, Boston, MA USA; 6 Department of Orthopaedic Surgery, Brigham & Women’s Hospital, Harvard Medical School, Boston, MA USA; 7 Neurovascular Pathophysiology Group, Centro Nacional de Investigaciones Cardiovasculares Carlos III (CNIC), Madrid, Spain

**Keywords:** Stroke, Astrocyte, Neuro-vascular interactions

## Abstract

The concept of the neurovascular unit emphasizes the importance of cell-cell signaling between neural, glial, and vascular compartments. In neurogenesis, for example, brain endothelial cells play a key role by supplying trophic support to neural progenitors. Here, we describe a surprising phenomenon where brain endothelial cells may release trans-differentiation signals that convert astrocytes into neural progenitor cells in male mice after stroke. After oxygen-glucose deprivation, brain endothelial cells release microvesicles containing pro-neural factor Ascl1 that enter into astrocytes to induce their trans-differentiation into neural progenitors. In mouse models of focal cerebral ischemia, Ascl1 is upregulated in endothelium prior to astrocytic conversion into neural progenitor cells. Injecting brain endothelial-derived microvesicles amplifies the process of astrocyte trans-differentiation. Endothelial-specific overexpression of *Ascl1* increases the local conversion of astrocytes into neural progenitors and improves behavioral recovery. Our findings describe an unexpected vascular-regulated mechanism of neuroplasticity that may open up therapeutic opportunities for improving outcomes after stroke.

## Abstract (web summary)

Damaged brains try to repair themselves by producing neurons in areas where neurogenesis does not normally occur. Here, the authors show that brain endothelial cells provide microvesicle-encased signals that convert parenchymal astrocytes into neural progenitors, thus improving outcomes after stroke.

## Introduction

Neurogenesis plays an important role in stroke recovery^1–3^. In models of cerebral ischemia, neurogenesis is amplified in the sub-ventricular zone (SVZ) and the sub-granular zone (SGZ)^4–6^. In addition to these responses in the standard neurogenic niches, there have also been reports that parenchymal astrocytes may convert into neurons via pathways dependent on Notch signaling^7–9^. But how this phenomenon is induced or regulated?

Within neurogenic niches in SVZ and SGZ, brain endothelial cells play a vital role by secreting trophic factors to support neural stem cells^10–12^. Within the broader concept of the neurovascular unit, endothelium is a central source of crosstalk signals that sustain homeostasis in adjacent neural and glial compartments^13^. The repertoire of brain endothelial signals is now recognized to include exosomes and extracellular vesicles that convey a wide range of functional messages to the parenchyma^14–16^.

In this work, we show that brain endothelial cells can release microvesicles that transfer the pro-neural transcription factor Ascl1 into astrocytes, thus converting them into neural progenitors. The significance and impact may be two-fold. First, this hypothesis would provide a non-cell autonomous mechanism for regulating cellular trans-differentiation. Second, the ability of brain endothelial cells to provide these endogenous trans-differentiation signals may lead unique therapeutic avenues for reprogramming the damaged brain.

## Results

### Oxygen-glucose deprivation-stimulated brain endothelial cells convert astrocytes into neural progenitor cells

**Fig. 1:**
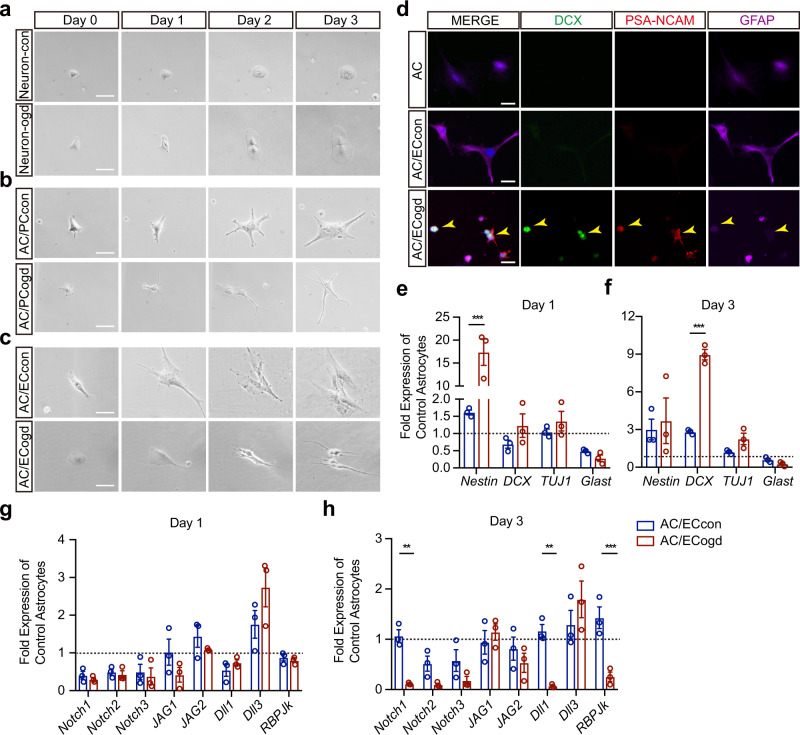
Oxygen-glucose deprivation-stimulated brain endothelial cells convert astrocytes into neural progenitor cells. **a** Morphologic changes of same astrocytes (AC) at day 0, day 1, day 2 and day 3 post cultured in conditioned neuron media collected from normal neurons (Neuron-con) or OGD-stimulated neurons (Neuron-ogd). **b** Morphologic changes of same AC at day 0, day 1, day 2 and day 3 post co-cultured with normal pericytes (PCcon) or OGD-stimulated pericytes (PCogd). **c** Morphologic changes of same AC at day 0, day 1, day 2 and day 3 post co-cultured with normal brain endothelial cells (ECcon) or OGD-stimulated brain endothelial cells (ECogd). **d** Representative immunostaining of DCX, PSA-NCAM and GFAP in AC or at day 3 post AC co-cultured with ECcon or ECogd. **e** mRNA levels of *Nestin*, *DCX*, *TUJ1* and *Glast* at day 1 post AC co-cultured with ECcon or ECogd; *n* = 3 biologically independent samples; *p* < 0.0001. **f** mRNA levels of *Nestin*, *DCX*, *TUJ1* and *Glast* at day 3 post AC co-cultured with ECcon or Ecogd; *n* = 3 biologically independent samples; *p* < 0.0001. **g** mRNA levels of Notch signaling-related genes at day 1 post AC co-cultured with ECcon or ECogd; *n* = 3 biologically independent samples. **h** mRNA levels of Notch signaling-related genes at day 3 post AC co-cultured with ECcon or ECogd; *n* = 3 biologically independent samples. *p* = 0.0077 for *Notch1*; *p* = 0.0017 for *Dll1*; *p* = 0.0007 for *RBP-Jκ*. Experiment was repeated independently 3 times with similar results (**a–d**). Data are shown as mean ± SEM. Two-way ANOVA with post-hoc Bonferroni adjustment. Scale bar, 50 μm. Source data are provided as a Source Data file.

To mimic the effects of stroke, we subjected astrocytes to 3 h of oxygen and glucose deprivation (OGD). Over the next 3 days, the astrocytes did not show any detectable shifts towards neuronal-like morphologies (Supplementary Fig. 1a). Therefore, it is likely that if astrocytes were to be reprogrammed after stroke, other cell types may provide non-cell autonomous signals for astrocyte trans-differentiation. We screened neurons, pericytes and brain endothelial cells as potential sources of trans-differentiation signals, by subjecting these cells to transient OGD and then either co-culturing them with astrocytes or transferring their conditioned media onto recipient astrocytes (Supplementary Fig. 1b). Brain endothelial cells but not neurons or pericytes, appeared to induce marked changes in co-cultured astrocytes (Fig. 1a–c). Co-culturing astrocytes with post-OGD brain endothelial cells but not normal brain endothelial cells changed the flat polygonal astrocytes into smaller round cells with bipolar processes, suggestive of a neuronal morphology (Fig. 1c). Immunostaining showed that these altered astrocytes expressed neural stem/progenitor markers (SOX2, PAX6, DCX and PSA-NCAM) by day 3 (Fig. 1d, Supplementary Fig. 1d, e). RT-PCR confirmed a shift from astrocyte to neural progenitor markers rapidly occur within the first 3 days (Fig. 1e, f). Thereafter, later marker like *Tuj1* begin to emerge from days 3–7 (Supplementary Fig. 2f), indirectly suggesting that these converted cells may continue to differentiate towards more mature cells over time. Notch signaling seems to be crucial in regulating the neurogenic ability of astrocytes, and blocking this signaling pathway by deletion of the gene encoding the obligate Notch coactivator *RBP-Jκ* triggers astrocyte trans-differentiation into neurons or neurogenic cells^8,9,17–19^. Therefore, we asked whether our phenomenon of brain endothelial-induced astrocyte trans-differentiation was consistent with these previously described Notch signals. After being co-cultured with OGD-stimulated brain endothelial cells but not normal brain endothelial cells, astrocytes showed a reduction in *Notch 1*, the Notch ligands *Dll1*, as well as *RBP-Jκ* (Fig. 1g, h). Finally, because standard astrocyte cultures are derived from neonatal rodents, there is a potential caveat that these findings were an artifact of immature astrocytes. We repeated experiments with adult rat astrocytes. Induction of neural progenitor markers were also observed in adult astrocytes after co-culture with post-OGD brain endothelial cells (Supplementary Fig. 1g).

### Microvesicles derived from OGD-stimulated brain endothelial cells reprogram astrocytes into neural progenitor cells

**Fig. 2:**
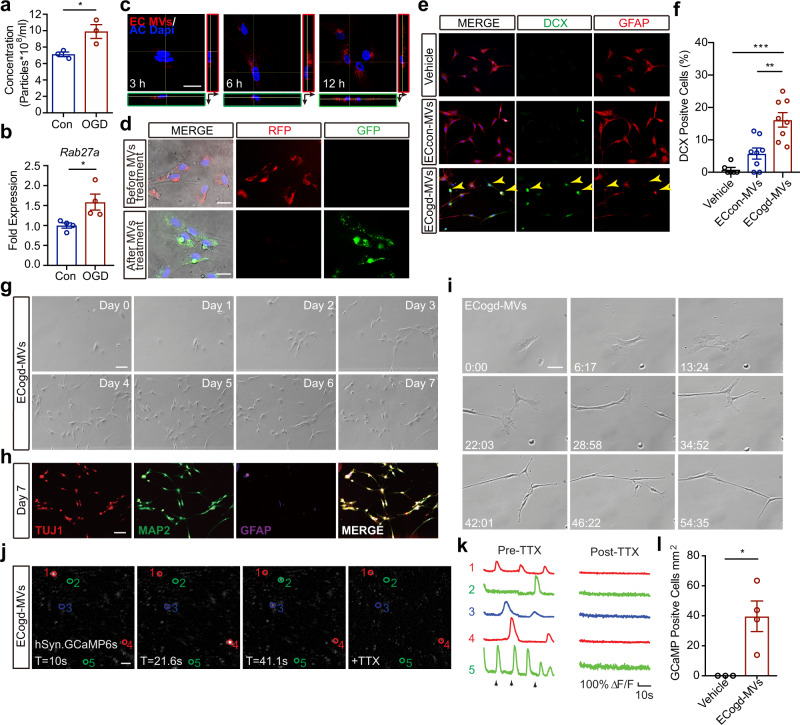
Microvesicles derived from OGD-stimulated brain endothelial cells reprogram astrocytes into neural progenitor cells. **a** Nanoparticle tracking analysis of microvesicles (MVs) derived from normal brain endothelial cells or OGD-stimulated brain endothelial cells; *n* = 3 biologically independent samples; *p* = 0.0354. **b** mRNA levels of *Rab27a* in normal brain endothelial cells or OGD-stimulated brain endothelial cells; *n* = 4 biologically independent samples; *p* = 0.0321. **c** Confocal microscopy revealed distribution of Cy3 labeled brain endothelial cells-derived MVs (EC-MVs) in AC at different time points. **d** Immunostaining images showing a red-to-green color switch of AC that had taken up Cre^+^ MVs derived from brain endothelial cells. **e** Representative immunostaining of DCX and GFAP in AC treated with vehicle or MVs derived from normal brain endothelial cells (ECcon-MVs) or OGD-stimulated brain endothelial cells (ECogd-MVs). **f** Quantification of DCX+ cells; *n* = 6 samples obtained from three independent experiments for vehicle group, *n* = 8 samples obtained from three independent experiments for ECcon-MVs group and ECogd-MVs group; Vehicle *vs* ECogd-MVs: *p* < 0.0001; ECcon-MVs *vs* ECogd-MVs: *p* = 0.0012. **g** Morphologic changes of same AC treated with ECogd-MVs at different time points. **h** Trans-differentiated cells in (**g)** were fixed at day 7 post ECogd-MVs treatment; Tuj1, Map2 and GFAP were detected by immunostaining. **i** Representative images of time-lapse live imaging analyses of individual AC after ECogd-MVs treatment. **j** Converted cells transduced with hSyn.GCaMP6S AAV and treated with 1 uM tetrodotoxin (TTX). **k** GCaMP6s traces for regions of interest 1–5; Arrows indicate the time points in (**j**). **l** Number of GCaMP+ cells in AC treated with vehicle or ECogd-MVs; *n* = 3 biologically independent samples for vehicle group, *n* = 4 biologically independent samples for ECogd-MVs group; *p* = 0.0217. Experiment was repeated independently 3 times with similar results (**c**, **d**, **g**–**j**). Data are shown as mean ± SEM. Unpaired two-tailed *t*-test (**a**, **b**, **l**); one-way ANOVA with post-hoc Tukey adjustment (**f**). Scale bar, 25 μm (**c**, **d**); 50 μm (**g**–**j**). Source data are provided as a Source Data file.

Extracellular microvesicles are emerging as novel mediators for endothelial-neural communication^14,15,20^. Therefore, we asked whether brain endothelial-induced trans-differentiation of astrocytes may involve microvesicle exchange. Microvesicles were purified from the extracellular culture medium of primary rat brain endothelial cell (Supplementary Fig. 2a–c). Nanoparticle analysis showed that brain endothelial-derived microvesicles were increased after OGD (Fig. 2a, Supplementary Fig. 2d, e) along with an increase in mRNA levels of *Rab27a* (Fig. 2b), a key regulator in microvesicle secretion^21^. Labeling microvesicles with Cy3 dye showed that they could be taken up by astrocytes over time (Fig. 2c). To confirm microvesicle uptake, we employed the Cre-LoxP system^22^ to induce a color switch in reporter-expressing astrocytes that take up microvesicles released from Cre recombinase-expressing brain endothelial cells (Supplementary Fig. 3a). Microvesicles isolated from Cre^+^ brain endothelial cells effectively induced a color switch in recipient astrocytes from RFP^+^ into GFP^+^ (Fig. 2d).

To assess functional relevance, we isolated microvesicles from brain endothelial cells after OGD and added them onto astrocytes, neurons and pericytes. Immunostaining suggested that converted astrocytes expressed neural progenitor markers at day 3, and showed evidence of proliferation (Fig. 2e, f, Supplementary Fig. 3b, c). In contrast, no significant effects were observed in neurons and pericytes (Supplementary Fig. 3d–f). Glial-like to neuronal-like morphologic changes were detected in recipient astrocytes from day 3 to day 7 (Fig. 2g). Concommitantly, neuronal markers (Tuj1, Map2) were increased at day 7 (Fig. 2h). No induction of neural shapes or markers were observed with vehicle or microvesicles from normal brain endothelial cells (Supplementary Fig. 3g–j). Time-lapse imaging confirmed the morphologic conversion of individual astrocytes after treatment with microvesicles from OGD-stimulated brain endothelial cells (Fig. 2i).

We next investigated whether these trans-differentiated cells showed neural-like function. After treatment with microvesicles from OGD-stimulated brain endothelial cells, the receipient astrocytes were transduced with an adeno-associated virus (AAV) that expressed GCaMP6s under the control of the neuronal synaptophysin promoter hSyn (hSyn.GCaMP6s)^23^. Calcium imaging^24^ showed activity in the trans-differentiated cells that resembled activity in “positive control” neurons, and these calcium responses were blocked by tetrodotoxin (TTX) treatment (Fig. 2j–l, Supplementary Fig. 3k, l, Supplementary Video 1 and 2). In contrast, we did not observe any activity in “negative control” astrocytes transduced with hSyn.GCaMP6s (Supplementary Video 3). The lack of calcium activity in astrocytes transduced with hSyn.GCaMP6s likely reflects the neuronal specificity of the hSyn promoter, since we observed slow calcium waves characteristic of astrocytes^25^ when expressing GCaMP6s using a ubiquitous promoter (pHAGE-RSV-GCaMP6s) (Supplementary Fig. 3m, n, Supplementary Video 4). Altogether, the neuronal specificity of the hSyn synaptophysin promoter, the change in calcium dynamics, and the blockade of responses by TTX, suggest that microvesicles from OGD-stimulated brain endothelial cells can indeed reprogram astrocytes into neural progenitor cells, and these progenitors may eventually show some potential of continuing onto cells with neuron-like calcium activity.

### Brain endothelial cell-derived microvesicles containing pro-neural transcription factor Ascl1 initiate astrocyte trans-differentiation

**Fig. 3:**
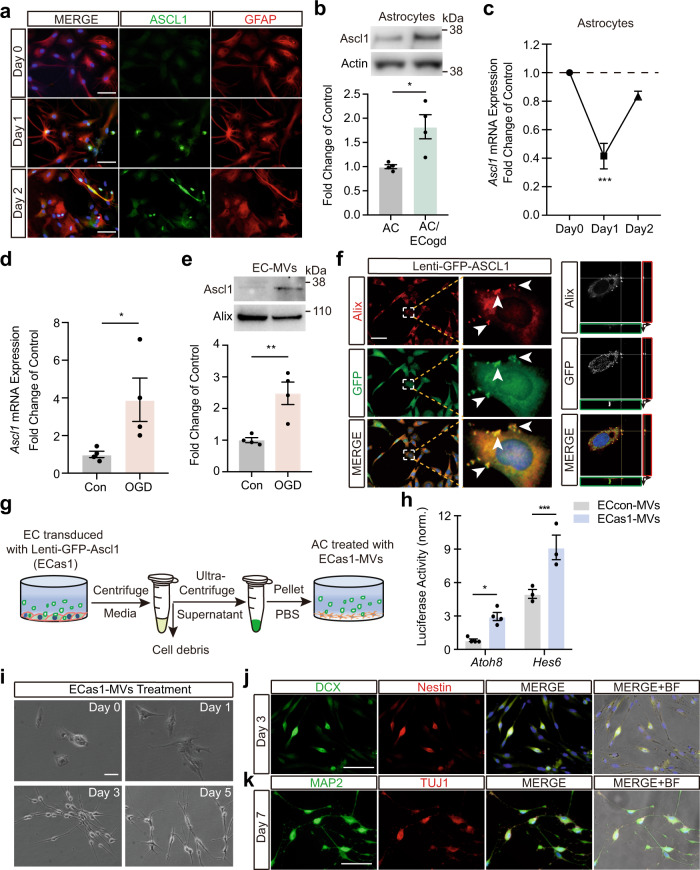
Brain endothelial cells-derived microvesicles containing pro-neural transcription factor Ascl1 initiate astrocytes trans-differentiation. **a** Representative immunostaining of Ascl1 and GFAP at day 0, day 1 and day 2 post AC co-cultured with OGD-stimulated brain endothelial cells. **b** Protein levels of Ascl1 in AC or AC at day 2 post co-cultured with OGD-stimulated brain endothelial cells (ECogd); *n* = 4 biologically independent samples; *p* = 0.0173. **c** mRNA levels of *Ascl1* in AC at day 0, day 1 and day 2 post co-cultured with OGD-stimulated brain endothelial cells; *n* = 3 biologically independent samples; *p* < 0.0001. **d** mRNA levels of *Ascl1* in normal or OGD-stimulated brain endothelial cells; *n* = 4 biologically independent samples; *p* = 0.0471. **e** Protein levels of Ascl1 in MVs derived from normal or OGD-stimulated brain endothelial cells; *n* = 4 biologically independent samples; *p* = 0.0065. **f** Confocal microscopy revealed co-localization of GFP and Alix in brain endothelial cells transduced with *GFP-Ascl1* lentivirus. **g** Experimental schematic for testing effects of MVs derived from brain endothelial cells overexpressed *Ascl1* (ECas1-MVs) on AC. **h** Luciferase activity showed increased activities of *Atoh8* and *Hes6* in AC treated with ECas1-MVs; *n* = 4 biologically independent samples for *Atoh8* group, *n* = 3 biologically independent samples for *Hes6* group; *p* = 0.0265 for *Atoh8;* *p* = 0.0009 for *Hes6*. **i** Morphologic changes of same AC at day 0, day 1, day 3 and day 5 post ECas1-MVs treatment. **j** Representative immunostaining of DCX and Nestin in AC at day 3 post ECas1-MVs treatment. **k** Representative immunostaining of Map2 and Tuj1 in AC at day 7 post ECas1-MVs treatment; BF: bright filed. Experiment was repeated independently 3 times (**a**, **f**, **i**–**k**) or 4 times (**b**, **e**) with similar results. Data are shown as mean ± SEM. Unpaired two-tailed *t*-test (**b**, **d**, **e**); one-way ANOVA with post-hoc Tukey adjustment (**c**); two-way ANOVA with post-hoc Bonferroni adjustment (**h**). Scale bar, 50 μm. Source data are provided as a Source Data file.

Cellular trans-differentiation is driven by specific transcription factors^26^. Ascl1, the pro-neural basic-helix-loop-helix (bHLH) fate-determination factor, is essential for neural reprogramming^27,28^. Therefore, we hypothesized that Ascl1 may be involved in this phenomenon of brain endothelial-driven astrocyte trans-differentiation. Astrocytes co-cultured with OGD-stimulated brain endothelial cells showed increased Ascl1 protein (Fig. 3a, b). Unexpectedly, however, the mRNA level of *Ascl1* in astrocytes was decreased at day 1 after co-culturing with OGD-stimulated brain endothelial cells (Fig. 3c). One possibility is that the increased Ascl1 protein within astrocytes may come from an exogenous source. Exosomes and extracellular vesicles are known to contain transcription factors^29–31^. Is it possible that Ascl1 is transferred into astrocytes from microvesicles released by OGD-stimulated brain endothelial cells?

Under normal conditions, brain endothelial cells expressed very low levels of *Ascl1* (Fig. 3d, Supplementary Fig. 4a). However, *Ascl1*, but not other neural transcriptional factors (*NeuroD1* and *Ngn2*), was upregulated after OGD (Fig. 3d, Supplementary Fig. 4a). Microvesicles purified from normal brain endothelial cells, pericytes, and astrocytes showed barely detectable levels of Ascl1 (Supplementary Fig. 4b). However, after OGD, only brain endothelial cells-derived microvesicle levels of Ascl1 were significantly increased (Fig. 3e, Supplementary Fig. 4b). To validate this phenomenon, we overexpressed *GFP*-tagged *Ascl1* in brain endothelial cells. GFP-Ascl1 signals were co-localized with the microvesicle marker Alix (Fig. 3f, Supplementary Fig. 4c). Notably, overexpressed *Ascl1* did not change the microvesicle release (Supplementary Fig. 4d). Topological experiments further confirmed that Ascl1 in the lumen of endothelial microvesicle by binding with the endosomal sorting complex required for transport-0 (ESCRT-0) subcomplex component HRS (Supplementary Fig. 4e, f). To show that brain endothelial microvesicle Ascl1 was functional, we constructed a luciferase-reporter system of *Hes6* and *Atoh8* which comprise two target genes of *Ascl1* that are activated during neural reprogramming^32^. When astrocytes were treated with microvesicles purified from *Ascl1* overexpressing brain endothelial cells (Fig. 3g), luciferase-reporter activities for *Hes6* and *Atoh8* were significantly increased (Fig. 3h). More importantly, Ascl1-containing microvesicles markedly shifted the morphology of astrocytes, and increased neural progenitor markers (DCX, Nestin) at day 3 and subsequently neuronal markers (Tuj1, Map2) at day 7 (Fig. 3i–k, Supplementary Fig. 4g). In contrast, Ascl1 siRNA decreased astrocyte trans-differentiation after treatment with post-OGD brain endothelial microvesicles (Supplementary Fig. 4h–j). Taken together, our findings demonstrate that brain endothelial cells that are stimulated with OGD can transfer Ascl1-containing microvesicles to initiate astrocyte trans-differentiation.

### Pro-neural transcription factor Ascl1 is expressed in brain microvessels after focal cerebral ischemia in mice

**Fig. 4:**
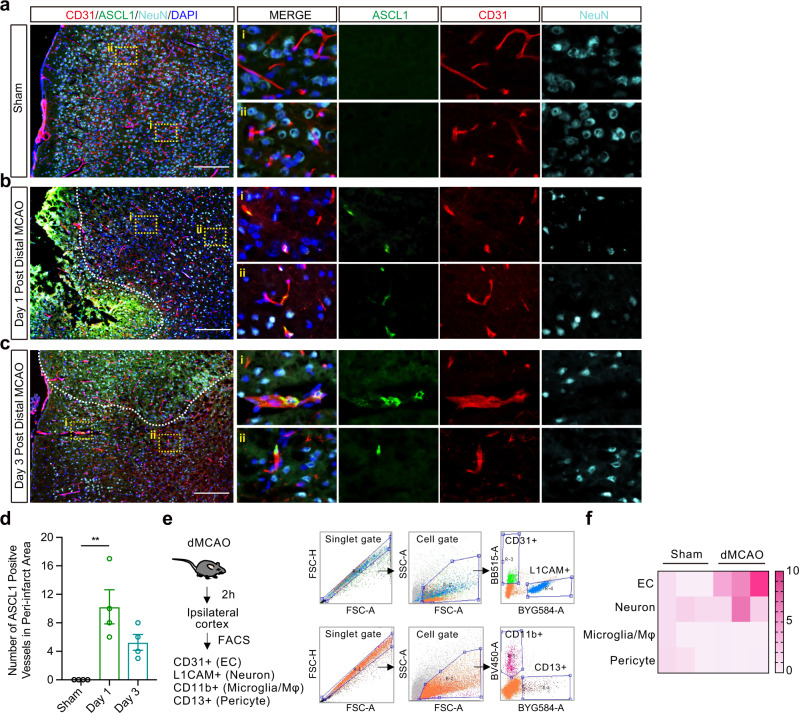
Pro-neural transcription factor Ascl1 is expressed in brain microvessels after focal cerebral ischemia in mice. Representative immunostaining of Ascl1, CD31, and NeuN at shame (**a**), day 1 (**b**), and day 3 (**c**) post permanent distal middle cerebral artery occlusion (MCAO); Dashed white line indicates lesion border. **d** Vessels were counted in 0.5 mm^2^ peri-infarct ROIs per section (totally 3 sections per mouse, 4 mice per group; Criteria is any loci with more than one Ascl1 positive cell); *p* = 0.0027. **e** Isolation of brain endothelial cell, neuron, pericyte, and microglia/Mφ from ipsilateral cortex by fluorescence-activated cell sorting (FACS) at 2 h post permanent distal MCAO. **f** mRNA levels of *Ascl1* in different cell isolated from (**e**). The experiment was repeated independently 4 times with similar results (**a–c**). Data are shown as mean ± SEM. One-way ANOVA with post-hoc Tukey adjustment. Scale bar, 200 μm. Source data are provided as a Source Data file.

If OGD is supposed to upregulate Ascl1 in brain endothelial cells, then we should be able to detect this response after stroke in vivo. Mice were subjected to permanent distal middle cerebral artery occlusion. In sham-operated controls, low level of Ascl1 was detected (Fig. 4a). At day 1 post-stroke, co-localizations of Ascl1 and the endothelial marker CD31 and the endothelial transcription factor ERG appeared within the ischemic boundaries (Fig. 4b, d, Supplementary Fig. 5a). To further verify the expression of Ascl1 in brain endothelial cells after stroke, microvessels were isolated from peri-infarct areas (Supplementary Fig. 5b). mRNA levels of *Ascl1* were increased in microvessels from ipsilateral hemisphere at 2 h compared to contralateral hemisphere (Supplementary Fig. 5c). At day 3 post-stroke, Ascl1 remained visible in brain endothelial cells (Fig. 4c, d). At day 7 post-stroke, Ascl1 began to be detectable in GFAP positive astrocytes (Supplementary Fig. 5d). To further assess Ascl1 expression in brain cells at the early stage after focal cerebral ischemia, brain endothelial cell, neuron, microglia/Mφ, and pericyte from ipsilateral cortex were isolated by fluorescence-activated cell sorting (FACS) at 2 h post permanent distal middle cerebral artery occlusion (Fig. 4e). Brain endothelial cells showed highest mRNA level of *Ascl1* after cerebral ischemia when compared to other cell types (Fig. 4f).

### Microvesicles derived from OGD-stimulated brain endothelial cells convert astrocytes into neural progenitor cells in mouse focal cerebral ischemia

**Fig. 5:**
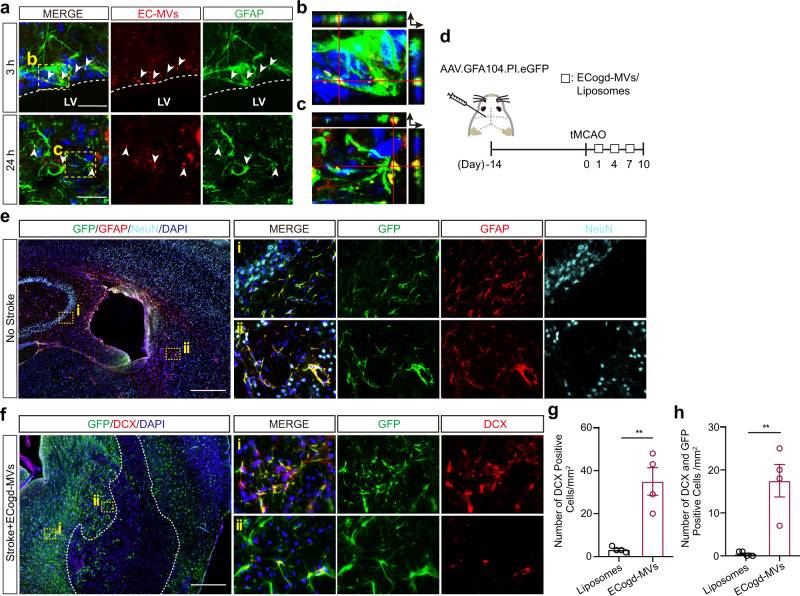
Microvesicles derived from OGD-stimulated brain endothelial cells convert astrocytes into neural progenitor cells in mouse focal cerebral ischemia. Confocal microscopy revealed Cy3 labeled primary brain endothelial cells-derived microvesicles (EC-MVs) could be taken into astrocytes at 3 h (**a**, **b**) and 24 h (**a**, **c**) post lateral ventricle injection; Dashed white line indicates lateral ventricle border. **d** Experimental design. **e** Representative immunostaining of GFP, GFAP, and NeuN at day 14 post lateral ventricle injection of AAV.GFA104.PI.eGFP. **f** Representative immunostaining of GFP and DCX at day 10 post 45 mins transient MCAO. **g** Number of DCX+ cells in 1 mm^2^ peri-infarct ROIs per section (totally 3 sections per mouse, 4 mice per group); *p* = 0.0026. **h** Number of both DCX+ and GFP+ cells in 1 mm^2^ peri-infarct ROIs per section (totally 3 sections per mouse, 4 mice per group); *p* = 0.0043. Experiment was repeated independently 3 times (**a**) or 4 times (**e**, **f**) with similar results. Data are shown as mean ± SEM. Unpaired two-tailed t-test. Scale bar, 25 μm (**a**); 200 μm (**e**, **f**). Source data are provided as a Source Data file.

Next, we sought to verify whether microvesicles derived from OGD-activated brain endothelial cells could stimulate astrocytes to undergo trans-differentiation in vivo. Microvesicles were purified from primary brain endothelial cells activated by OGD, then injected into the lateral ventricles of C57Bl6 mice. Immunostaining suggested astrocytes can take up brain endothelial-derived microvesicles (Fig. 5a–c, Supplementary Fig. 6a); co-localization of labeled microvesicles with astrocytes were observed around the SVZ at 3 h after injection (Fig. 5a, b). Besides SVZ, this co-localization could also be detected in striatum at 24 h after injection (Fig. 5a, c). To further improve the ability to track astrocyte trans-differentiation, we injected an AAV that expresses *eGFP* under the control of the astrocyte promoter *GFAP* (*GFA104-eGFP*)^33^ into the lateral ventricle to label astrocytes (Fig. 5d). Immunostaining showed effective co-localization of GFP with GFAP signals (Fig. 5e, Supplementary Fig. 6b, c). Mice were then subjected to transient occlusion of the middle cerebral artery and treated with OGD-stimulated brain endothelial microvesicles or control liposomes (Fig. 5d). At day 10 post-stroke, co-localizations of GFP and neural progenitor markers (DCX, PSA-NCAM) were observed in peri-infarct striatum in microvesicle-treated mice (Fig. 5f, Supplementary Fig. 6d, e). More importantly, either DCX-positive cells or both DCX-positive and GFP-positive cells were significantly increased in microvesicle-treated mice (Fig. 5g, h). Recently, the tamoxifen-inducible *Aldh1l1-CreER*^*T2*^*;R26R-YFP* line has been reported to be superior for tracing the lineage of astrocytes with minimal labeling of endogenous neurons^34^. Astrocyte labeling specificity was confirmed in the tamoxifen-treated adult *Aldh1l1-CreER*^*T2*^*;R26R-YFP* mouse (Supplementary Fig. 7a–c). We then injected OGD-stimulated brain endothelial microvesicles or liposomes into the lateral ventricle of middle cerebral artery occluded mice (Supplementary Fig. 7d). At 10 days post-occlusion, increased DCX or PSA-NCAM positive cells were labeled with YFP in peri-infarct striatum in the microvesicle-treated group (Supplementary Fig. 7e, f), consistent with the results in wild-type mice (Fig. 5f–h, Supplementary Fig. 6d, e). Thus, our data suggest that microvesicles derived from OGD-stimulated brain endothelial cells may convert astrocytes into neural progenitor cells after stroke in vivo.

### Overexpression of Ascl1 in brain endothelial cells increases neurogenesis and improves neurological recovery after focal cerebral ischemia

**Fig. 6:**
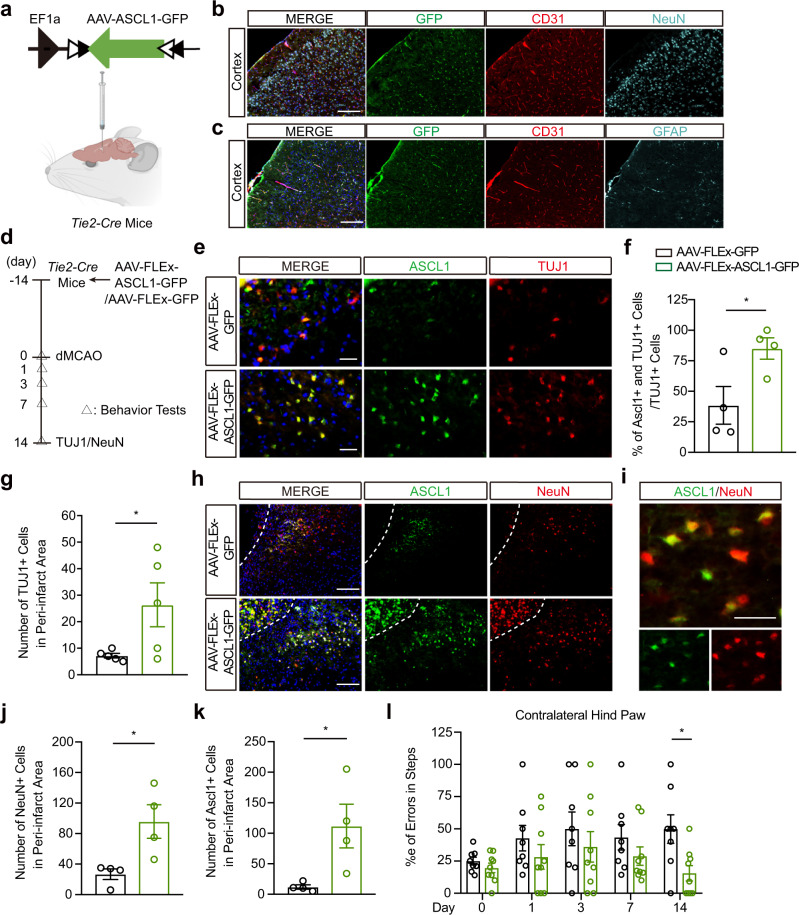
Overexpression of Ascl1 in brain endothelial cells increases neurogenesis and improves neurological recovery after focal cerebral ischemia. **a** AAV-FLEx-ASCL1-GFP or AAV-FLEx-GFP was injected into lateral ventricle of the *Tie2-cre* mice. Created with BioRender.com. **b** Representative immunostaining of GFP, CD31 and NeuN in cortex at day 8 post AAV-FLEx-ASCL1-GFP injection. **c** Representative immunostaining of GFP, CD31 and GFAP in cortex at day 8 post AAV-FLEx-ASCL1-GFP injection. **d** Experimental design. **e** Representative immunostaining of Ascl1 and TUJ1 at day 14 post permanent distal MCAO. **f** Quantification of TUJ+ cells expressing *Ascl1* in the peri-infarct area (totally 3 sections per mouse, 4 mice per group); *p* = 0.0392. **g** Quantification of TUJ1+ cells in the peri-infarct area (totally 3 sections per mouse, 5 mice per group); *p* = 0.0496. **h** Representative immunostaining of Ascl1 and NeuN at day 14 post distal permanent MCAO; Dashed white line indicates lesion border. **i** Representative immunostaining of Ascl1 and NeuN at day 14 post distal permanent MCAO after AAV-FLEx-ASCL1-GFP injection. **j**–**k** Quantification of NeuN+ cells (**j**) and Ascl1+ cells (**k**) in the peri-infarct area (totally 3 sections per mouse, 4 mice per group); *p* = 0.0245 in (**j**); *p* = 0.0323 in (**k**). **l** Foot fault test (*n* = 8 mice for AAV-FLEx-GFP group, *n* = 9 mice for AAV-FLEx-ASCL1-GFP group); *p* = 0.0439. The experiment was repeated independently 5 times with similar results (**b, c**). Data are shown as mean ± SEM. Unpaired two-tailed *t*-test (**f**, **g**, **j**, **k**); two-way ANOVA with post-hoc Bonferroni adjustment (**l**). Scale bar, 25 μm (**e**, **i**); 100 μm (**b, c**, **h**). Source data are provided as a Source Data file.

Finally, if brain endothelial cells can transfer Ascl1 to induce astrocyte trans-differentiation, can one augment this phenomenon to increase neurogenesis and modify outcomes after stroke? To assess this idea, we used AAVs to express *Ascl1* with inverted orientation and flanked by two pairs of loxP. This allows the expression of *Ascl1* specifically in endothelial cells when injecting transgenic mice expressing *Cre* recombinase under either the *Tie2* or *VE-cadherin* promoter (*Tie2-Cre* or *VE-cadherin-Cre* mice) (Fig. 6a, Supplementary Fig. 8b). We observed robust co-localization of GFP with endothelial cell marker (CD31) in cortex at 8 days post-injection (Fig. 6b, c). Accordingly, less than 5% GFP positive cells were positive for non-endothelial markers, such as NeuN (Supplementary Fig. 8a). In order to analyze the effects of brain endothelial overexpressing *Ascl1* on neurogenesis after stroke, *Tie2-Cre* and *VE-cadherin-Cre* mice were injected with AAV-FLEx-GFP or AAV-FLEx-ASCL1-GFP and then subjected to permanent occlusion of their distal middle cerebral arteries (Fig. 6d, Supplementary Fig. 8c). By 14 days post-stroke, the percentage of TUJ1 positive cells expressing *Ascl1* and the number of TUJ1 positive cells were significantly higher after endothelial-specific *Ascl1* upregulation (Fig. 6e–g, Supplementary Fig. 8d–f). Moreover, NeuN positive neurons were significantly increased (Fig. 6h–j), accompanied by more Ascl1 positive cells in mice with endothelial-specific *Ascl1* upregulation, compared to the control virus group (Fig. 6k). We then asked whether brain endothelial Ascl1-driven neurogenesis was associated with stroke recovery. In the foot-fault test, *Tie2-Cre* mice treated with AAV-FLEx-ASCL1-GFP showed significantly fewer errors by day 14 compared to AAV-FLEx-GFP treated mice (Fig. 6l). In the tape-removal test, *VE-cadherin-Cre* mice treated with AAV-FLEx-ASCL1-GFP showed significantly faster tape removal (day 14 *vs* day 1) (Supplementary Fig. 8g). All these data suggest that endothelial-specific overexpression of *Ascl1* may contribute to neurogenesis and partly improve neurological recovery after stroke.

## Discussion

In adult mammalian brains, baseline neurogenesis is typically restricted to the SVZ of the forebrain and SGZ of the hippocampus which are the major niches that harbor neural stem cells. However, it has been hypothesized that parenchymal astrocytes after injury may potentially convert into neural stem cells since they share expression of several biomarkers^35^. Astrocyte-into-neuron conversion can be detected in mice subjected to focal cerebral ischemia or excitotoxic lesions^8,36^. What has remained unclear is how this phenomenon is induced or regulated. Our study shows that a non-cell autonomous mechanism may be involved, with brain endothelial cells releasing microvesicles that transfer the pro-neural transcription factor Ascl1 into astrocytes, thus converting them into neural progenitors.

Many intra-cellular phenomena are regulated by inter-cellular signals. At the blood-brain barrier, the intra-cellular organization of endothelial tight junctions and transporters require inter-cellular signals from adjacent astrocytes and pericytes^37^. For multi-cellular organisms, the mechanisms of tolerance and pre-conditioning are augmented by help-me signals that exchange between individual cells^38–40^. And as pointed out before, the classic neurogenic niche requires endothelial signals to support neural stem cells^10,11^. Our study suggests that brain endothelium can do even more, i.e. not only provide growth factors for stem cells, but also act as a potential source of microvesicle signaling that regulates the delicate process of trans-differentiation within the brain.

In the context of stroke, astrocytes play a significant but complex role in brain recovery^41,42^. On one hand, reactive astrocytes can be detrimental by forming “glial scars” that inhibit neuronal rewiring^43^. Alternatively, some phenotypes of activated astrocytes can be beneficial by supporting neuroplasticity^44^ and angiogenesis^45^. Hence, ongoing efforts are growing to find small molecules that may target astrocyte response in central nervous system (CNS) injury and disease^42^. Our findings here may offer a different approach. This ability of brain endothelial microvesicles to reprogram astrocytes into neural progenitors may provide vascular avenues for manipulating glial responses in stroke therapy. Our discovery of this endogenous pathway may significantly extend the concept and utility of extracellular microvesicles for cell reprogramming^30^.

Nevertheless, there are a few caveats. First, it is known that stroke can alter the profile of microvesicles synthesized and released from endothelial cells and these microvesicles can pass through the blood-brain barrier into brain^46^. However, the contents of these microvesicles (including proteins and nucleic acids) may differ depending on injury severity^47^. In our experimental systems, brain endothelial cells were subjected to mild OGD. Whether brain endothelial cells damaged by more severe OGD have different effects on astrocyte trans-differentiation should be examined. Second, extracellular microvesicles can contain many other non-secreted proteins and DNA/RNA^46,48^. We focused on Ascl1 because it is a primary transcription factor for neural reprogramming. However, other factors such as RNA-binding protein PTB have been demonstrated to convert both mouse and human astrocytes to functional neurons^49,50^. How multiple extracellular microvesicle signals contribute to neurogenesis is an area for further investigation. Third, although forced expression of *Ascl1* alone or with other factors can regenerate both glutamate and GABAergic neurons^27,28,51^, our study is focused on the ability of brain endothelial signals to trans-differentiate astrocytes into neural progenitors and improve outcomes after stroke. Further studies are required to assess full maturation of these neural progenitors and determine what classes of neurons need to be replaced in damaged or diseased brain to recover clinical function. Fourth, astrocytes play complex and diverse roles in the remodeling CNS^52–54^. Further investigations are needed to define different subsets of astrocytes that can be effectively reprogrammed after injury. Finally, the regional environment and intrinsic lineage of neighboring cells may be a critical factor in sustaining cell identity. The addition of exogenous transcription factors induces fewer new neurons in the striatum compared to cortex^55^. In *RBP-Jκ*-deficient mice, unlike striatum, astrocytes in the cortex fail to complete the neurogenic program that they initiate^19^. Thus, it will be important to understand how subtle regional differences may influence the brain endothelial regulation of astrocyte reprogramming after stroke.

In summary, we identified a non-cell autonomous mechanism for trans-differentiation, wherein brain endothelial cells induce the conversion of astrocytes into neural progenitors. This vascular-regulated form of neuroplasticity may open up opportunities for pro-recovery therapies after stroke.

## Methods

### Primary rat brain endothelial cells/Primary pericytes and Primary rat astrocytes co-cultures

Rat brains were collected in ice-cold sterile Phosphate Buffered Saline (PBS). Meninges were removed. Gray matter was cut into 1 mm^3^ pieces and digested with 1 mg/ml collagenase CLS2 (Worthington Biochem., Lakewood, NJ, USA) in DMEM at 37 °C for 1.5 h. Microvessels were isolated by centrifugation (1000 × *g*, 20 min) in 20% BSA-DMEM and further digested with 1 mg/ml collagenase-dispase (Roche, Basel, Switzerland) in DMEM for 1 h. Brain microvascular endothelial cells (EC) were collected in a 33% continuous Percoll gradient (1000 × *g*, 10 min), and then washed twice in DMEM. Isolated EC were seeded on collagen type IV-coated plates. Cells were maintained in endothelial basal medium-2 (Lonza, Hopkinton, MA) supplemented with fetal bovine serum (FBS), fibroblast growth factor-2, epidermal endothelial growth factor, hydrocortisone, insulin-like growth factor, ascorbic acid, vascular endothelial growth factor, and amphotericin B. During the first 2 days, culture medium contained puromycin (4 μg/mL) to selectively remove P-glycoprotein-negative contaminating cells.

Primary astrocyte cultures (AC) were prepared from the cerebral cortices of 1-day-old neonatal Sprague-Dawley rats. Briefly, the dissociated cortical cells were suspended in DMEM containing 10% FBS and plated in 75-cm^2^ flasks. After 10–14 days in vitro, AC were obtained from the mixed glial cultures by shaking at 220 rpm overnight to remove microglia and oligodendrocytes. AC were dissociated by trypsinization and then reseeded on collagen type IV-coated plates. Passages 2–4 of primary cultured AC were used for experiments.

Human brain vascular pericytes (PC) were purchased from ScienCell (#1200) and cultured in pericyte medium containing 2% FBS and pericyte growth supplement (ScienCell).

EC or PC were cultured on the collagen-coated trans-well membrane at a density of 5 × 10^5^ cells per ml. After 24 h, EC or PC were subjected to 3 h OGD or control non-OGD condition. The trans-well membrane with EC or PC monolayer was placed on top of the AC immediately after pre-exposure to OGD or non-OGD, at a density of 2 × 10^3^ cells per ml for AC. The EC/AC or PC/AC co-culture was maintained at 37 °C for 1–7 days. Medium was changed every other day. AC were fixed or collected and processed for immunohistochemistry, Western-blot and quantitative PCR analyses as described below.

### Conditioned medium (CM) of neurons preparation

Primary neuron cultures were prepared from the corticies of embryonic day (E)17 rat embryos (Charles River Laboratory). Briefly, the cortical tissues were dissected and digested with Trypsin (Invitrogen). The cells were then plated onto poly-D-lysine-coated plates at a density of 3 × 10^5^ cells/ml and cultured in DMEM (Life Technology, 11965-084) containing 5% FBS. After 24 h, the medium was changed to NeuroBasal medium (Invitrogen, 21103-049) supplemented with 2% B-27 (Invitrogen, 17504044), 0.5 mM L-glutamine and 1% penicillin-streptomycin and replaced every 2–3 days. The neuron cells were cultured in humidified incubator at 37 °C and 5% CO_2_ and were used for experiments from 8 to 11 days in vitro. For preparation of CM for treating AC, neurons were incubated in medium for 24 h, with or without OGD, and CM was collected. AC were treated with neuronal CM and AC medium at a ratio of 1:1. Medium was changed every other day.

### OGD and reoxygenation

To induce OGD, the culture medium was replaced with deoxygenated, glucose-free DMEM (Life Technology, 11966-025) and cells were placed in a humidified chamber (Heidolph, incubator 1000, Brinkmann Instruments) which was perfused with anaerobic gas mixture (90% N_2_, 5% H_2_, and 5% CO_2_) for 30 min, then sealed and kept at 37 °C. After 3 h of OGD, cultures were removed from the anaerobic chamber, and the medium was replaced with EndoGRO Basal Medium (Millipore, SCME-BM) with or without the growth factors. Cells were then allowed to recover for 24 h for cell viability assay in a regular incubator.

### Immunohistochemistry

Samples were initially fixed with 4% paraformaldehyde for 10 min at room temperature. Then, samples were processed with 0.1% Triton X for 5 mins, followed by 5% Bovine serum albumin blocking for 1 h at room temperature. After staining with primary antibody overnight at 4 °C (Anti-Ascl1 antibodies, 1:100, Thermo Fisher Scientific, 14579482; anti-CD31 antibody, 1:100, BD Biosciences, 565629; anti-NeuN antibody, 1:200, Millipore, MAB377; anti-doublecortin antibody, 1:100, Cell Signaling Technology, 4604; anti-Nestin antibody, 1:100, Abcam, ab11306; anti-GFAP antibody, 1:200, Innovative Research, 13-0300; anti-MAP2 antibody, 1:100, Abcam, ab32454; anti-TUBB3 antibody, 1:100, BioLegend, 801209; anti-GFP antibody, 1:100, Santa Cruz Biotechnology, sc-9996; anti-Alix antibody, 1:100, Cell Signaling Technology, 2171; anti-PSA-NCAM, 1:100, Thermo Fisher Scientific, 14911882; anti-SOX2 antibody, 1:200, Millipore, AB5603; anti-PAX6 antibody, 1:200, Thermo Fisher Scientific, 13B10-1A10; anti-Ki67 antibody, 1:200, Abcam, ab15580). After washing off the primary antibody with PBS, the samples were then incubated with secondary antibodies (1:200; Jackson Immunoresearch Laboratories) for 1 h at room temperature. After that, the samples were covered with VECTASHIELD with DAPI (Vector Laboratories). Immunostaining was analyzed with a fluorescence microscope (Nikon).

### Quantitative PCR analysis

Total RNAs were extracted using QIAzol lysis reagent (QIAGEN, 79306) from cells, followed by cDNA synthesis using High-capacity RNA-to-cDNA kit (ThermoFisher Scientific, 4387406) according to the manufacturer’s instructions. Relative levels of genes were determined by amplifying *TUBB3* (Applied Biosystems, Rn01431694_m1), *DCX* (Applied Biosystems, Rn00670390_m1), *GFAP* (Applied Biosystems, Rn01253033_m1), *GLAST* (Applied Biosystems, Rn01402419_g1), *NESTIN* (Applied Biosystems, Rn01455599_g1), *NOTCH1* (Applied Biosystems, Rn01758633_m1), *NOTCH2* (Applied Biosystems, Rn01534371_m1), *NOTCH3* (Applied Biosystems, Rn00571731_m1), *JAG1* (Applied Biosystems, Rn00569647_m1), *JAG2* (Applied Biosystems, Rn00439932_m1), *DLL1* (Applied Biosystems, Rn00583276_m1), *DLL3* (Applied Biosystems, Rn00586568_m1), *RBPJ* (Applied Biosystems, Rn01457015_g1), *ASCL1* (Applied Biosystems, Rn00574345_m1, Mm03058063_m1), *Rab27a* (Applied Biosystems, Rn00568995_m1, Mm00469997_m1), *Myt1l* (Applied Biosystems, Rn00485418_m1), *Neurod1* (Applied Biosystems, Rn00824571_s1), *Neurog2* (Applied Biosystems, Rn07363986_s1), and normalized by housekeeping gene *B2M* (Applied Biosystems, Rn00560865_m1, Mm00437762_m1).

### Microvesicle isolation and analyses

1*10^7^ brain endothelial cells were cultured in exosome-free medium for one day, after which the medium was collected, centrifuged to remove cells, immediately followed by microvesicles (MVs) isolation in 4 °C. MVs were collected by Total Exosome Isolation Reagent (ThermoFisher Scientific, 4478359) or ultracentrifugation^56^: supernatant fractions collected from cell cultures were pelleted by centrifugation at 500 × g for 10 min. The supernatant was centrifuged at 20,000 × *g* for 20 min. MVs were then harvested by centrifugation at 100,000 × *g* for 70 min. The MVs pellet was resuspended in 20 ml of PBS and collected by ultracentrifugation at 100,000 × *g* for 70 min (Sorvall Surespin 630 rotor). Total protein concentration of MVs was measured by Pierce Coomassie (Bradford) Protein Assay Kit (ThermoFisher). For in vitro experiments, astrocytes were treated with MVs purified from cultured brain endothelial cells at concentration of 500 ng/ml. For in vivo experiments, MVs treatment (1 μg protein) was performed by using MVs purified from cultured brain endothelial cells three times for a duration of 10 days as indicated.

### Nanoparticle tracking analysis

Extracellular vesicles were purified by ultracentrifugation or kit and quantified using the Nanosight LM10 (Malvern, Framingham, MA)^57^. Samples were diluted in PBS 1× (1:20) from the concentrated pellet. Each sample was recorded three times for 30 s, with manual monitoring of temperature and cameral level set to 14. Analysis was performed using the NTA v3.1 software, with detection threshold set to 7 (Malvern, Framingham, MA). NTA extracellular vesicles concentration was expressed in extracellular vesicles/ml and extracellular vesicles size in mean values.

### Microvesicles labeling

Primary rat brain endothelial cells-derived microvesicles were labeled by using ExoGlow-Membrane EV Labeling Kit (SBI, EXOGM600A-1). Briefly, 50–100 μg microvesicles were added into the labeling reaction buffer and incubated at room temperature for 30 min. Free unlabeled dye was removed by PD SpinTrap G-25 (GE Healthcare, 28-9180-07).

### Calcium imaging

Cells were transduced with hSyn.GCaMP6s AAV (Addgene, 100843) or pHAGE-RSV-GCaMP6s lentivirus (plasmid was purchased from Addgene (80146) and packaged by the MGH viral core) at 5 days before calcium imaging. Converted cells were imaged between 18 and 21 days after initial microvesicles treatment by ImageXpress micro-confocal at 10 hz in widefield mode. MATLAB and ImageJ were used for data acquisition and analyses.

### Western-blot

Protein samples were loaded onto 4–12% Tris-glycine gels. After electorophoresis and transferring to nitro-cellulose membranes, the membranes were blocked in Tris-buffered saline containing 5% no-fat milk for 60 min at room temperature. Membranes were then incubated overnight at 4 °C with anti-Ascl1 antibody (1:100, Santa Cruz Biotechnology, sc-374104), anti-CD63 antibody (1:200, Santa Cruz Biotechnology, sc-5275), anti-Alix antibody (1:1000, Cell Signaling Technology, 2171), anti-TSG101 antibody (1:200, Santa Cruz Biotechnology, sc-7964), anti-ApoA1 antibody (1:200, Santa Cruz Biotechnology, sc-69755), anti-HRS antibody (1:1000, Santa Cruz Biotechnology, sc-271455), anti-Occludin antibody (1:500, Invitrogen, 71-1500), anti-Claudin-5 antibody (1:1000, Invitrogen, 35-2500) and anti-β-actin antibody (1:2000, Sigma-Aldrich, A5441). After incubation with peroxidase-conjugated secondary antibodies, visualization was enhanced by chemiluminescence (GE Healthcare, NA931- anti-mouse, or NA934- anti-rabbit). Optical density was assessed using the ImageJ. Uncropped versions of the blots are shown in the Source Data.

### Luciferase-reporter system

Rat primary astrocytes whole genomic DNA was extracted by the DNA extraction kit (IBI, IB47202). PCR reactions were performed to amplify the promoter of *HES6* and *Atoh8* with the following primers: *HES6*-fwd: GTAGTCTACGGTACCGGGAGAAGTACAACACAGTT; *HES6*-rev: TATTCTAATAGCTCGAGTCCGCCCCGTGGGGCCCCGC; *Atoh8*-fwd: AATCTTGAAGGTACCCCAGGGGGCGCTGCCCAGCT; *Atoh8*-rev: ATAGTCATAAGCTTGGCGCGGACGGCGCGAGGCA. The size of amplified fragments was 2000 bp. After being confirmed by Sanger DNA sequencing, the obtained DNA fragments from *Atoh8* promoter were ligated into the Kpn I and Hind III restriction enzyme sites of pGL4.10 plasmid (Promega, E665A). The obtained DNA fragments from *HES6* promoter were ligated into the Kpn I and XHO I restriction enzyme sites of pGL4.10 plasmid. The recombinant plasmid was tested and sequenced with primer *RVP-3*: TAGCAAAATAGGCTGTCCC. The positive cloned plasmid was used for cell transfection. Primary rat endothelia cells with *Ascl1* overexpression were chosen as target cells for the follow-up study. These cells were vaccinated in a 24-well culture plate, and when these cells had grown to 80–90%, 800 ng of pGL3 recombinant plasmid and 10 ng of phRL-SV40 were transfected according to the instructions of Lipofectamine 2000 liposomes (ThermoFisher Scientific, 11668019). While establishing pGL3-Enhancer as a negative control and pGL3-Control as a positive control for transfection of 30 h, luciferase-reporter gene activity was detected following dual luciferase assay kit steps (Promega, E1910). The culture solution was discarded and the cells were washed with cold PBS twice. Then 100 μl of passive lysis buffer (PLB) was added to each well, and it was placed on a shaker at room temperature for slow shaking for 15–20 min. After repeated freezing and thawing, cell lysates were transferred to a 0.5 ml centrifuge tube for brief centrifugation of 10 s. Then 20 μl of cell lysate was added to 100 μl of fluorescence luciferase substrate (LAR II), and liquid scintillation luciferase was measured for the luminescence value after mixing. Then 100 μl of reaction terminated liquid (Stop & Glo Reagent) was added and mixed for measurement of internal standard Renilla luciferase with a liquid scintillation counter. The ratio of the two is the relative luciferase activity (RLA). Each recombinant plasmid and control plasmid were transfected and detected three times.

### Animals

Since female mice must be tested across the estrous cycle and are more variable than males, male mice were used in this proof-of-concept study. Female and male mice will be included in our future studies. C57BL6 (Jax Stock No: 000664) mice, (B6.Cg-Tg(*Tek-cre*)1Ywa/J (Jax Stock No: 008863) mice, B6.FVB-Tg(*Cdh5-cre*)7Mlia/J (Jax Stock No: 006137) mice, *Aldh1l1- CreER*^*T2*^ (Jax Stock No: 029655), and *R26R-YFP* (Jax Stock No: 006148) were obtained from the Jackson Laboratory^34,58–60^. Twelve-week males of mouse lines were used for experiments. All mice were (up to 4 mice per cage) maintained in a controlled pathogen-free/germ-free environment with a temperature of 68–73 °F, 12/12 h light/dark cycle, 30–70% humidity, and food (Prolab Isopro RMH3000 Irradiated, 3003219-249) and water provided ad libitum. Mice were anesthetized using isoflurane (2–3% induction, 1–1.5% maintenance), and received buprenorphine (0.05–0.1 mg/kg, intraperitoneal injection) pre- and post-surgery. Mice were deeply anesthetized with isoflurane (3%), and then subjected to cardiac perfusion before brain removal. All experiments were performed under approved Institutional Animal Care and Use Committee protocols (2010N000147 and 2016N000493) in accordance with National Institutes of Health guidelines and with the United States Public Health Service’s Policy on Human Care and Use of Laboratory Animals and following Animals in Research: Reporting In vivo Experiments (ARRIVE) guidelines.

For tamoxifen administration, tamoxifen (Sigma) was dissolved in a mixture of ethanol and sesame oil (1:9 by volume) at a concentration of 40 mg/ml^34^. Tamoxifen was administered through intraperitoneal injections at a daily dose of 1 mg/10 g body weight for 5 days.

### Distal permanent MCA occlusion

Ascl1 expression and the effect of endothelial-specific *Ascl1* overexpression on neurogenesis after ischemic stroke were assessed in a distal permanent MCA occlusion model. A small craniotomy was made over the trunk of the left MCA and above the rhinal fissure. Distal permanent MCA occlusion was performed by ligature of the trunk just before its bifurcation between the frontal and parietal branches with a 9-0 suture, in combination with the occlusion of the ipsilateral common carotid artery. Complete interruption of blood flow was confirmed under an operating microscope. These experimental conditions led to moderately sized cortical infarcts. Rectal temperature was maintained at 37 °C with a thermostat-controlled heating pad. Following surgery, individual animals were returned to their cages with free access to water and food.

### Flow cytometry analysis

Tissues collected from ipsilateral hemisphere were gently minced and then digested at 37 °C for 30 min with an enzyme cocktail (Collagenase type IV; Sigma-Aldrich, C5138, DNase I; Sigma-Aldrich, D4263). FACS analysis was performed using a no labeled control for determining appropriate gates, voltages, and compensations required in multivariate flow cytometry. Data was assessed using the FlowJo software.

### Transient occlusion of the MCA

The effect of brain endothelial cells-derived microvesicles on astrocytes trans-differentiation after ischemic stroke was assessed in a transient occlusion of the MCA model. Transient focal ischemia was induced introducing a 6-0 (in mice) surgical monofilament nylon suture (Doccol) from the right external carotid artery into the internal carotid artery and advancing it to the branching point of the right MCA. Adequate ischemia was confirmed by continuous laser Doppler flowmetry (LDF) (Perimed, North Royalton, OH, USA). Animals that did not have a significant reduction to less than 30% baseline LDF values during MCA occlusion were excluded. After occluding the MCA for 45 min, the monofilament suture was gently withdrawn in order to restore blood flow. Rectal temperature was maintained at 37 °C with a thermostat-controlled heating pad. Following surgery, individual animals were returned to their cages with free access to water and food.

### Brain microvessels isolation

The brain microvessels were extracted from the cortex of mice, as in our previous report^61^. Briefly, mice were anesthetized by isofluorane and perfused with 40 ml HBSS (Invitrogen). The cerebral cortex was dissected and after removing surface large blood vessels, tissue was homogenized in cold PBS on ice with Knote Dounce glass tissue grinder (Part 885300-0002; Kimble Chase Life Science, Vineland, NJ), then centrifuged at 4 °C, 500 × *g* for 5 min. The tissue pellet was suspended with 18% Dextran solution (molecular weight 60–90 kDa; USB Corporation, Cleveland, OH) in PBS and then centrifuged again at 4 °C, 2500 × *g* for 20 min. The suspension was filtered through a 40-μm cell strainer to get rid of remaining single cells or small cell clumps. The resulting microvessels on the top of the cell strainer were used directly for further analysis.

Adeno-associated virus vector construction and virus production pAAV.GFA104.PI.eGFP.WPRE.bGH was a gift from Philip Haydon (Addgene viral prep # 100896-AAV5; http://n2t.net/addgene:100896; RRID:Addgene_100896). Cre-dependent AAV viral vectors were constructed based on AAV-pEF1α-FLEX -WPRE-pA plasmid (gift from Jie Xu in Kong Lab, Tufts University). PCR reactions were performed to introduce silent mutations in *Asc1* sites from Tet-O-FUW-Ascl1 (Addgene, 27150)) with the following primers: ci*ASCL1*-fwd: CAGCCGGCCGCCGTGGCGCGACGCAACGAGCGCGAGCGCAAC and ci*ASCL1*-rev: GTTGCGCTCGCGCTCGTTGCGTCGCGCCACGGCGGCCGGCTG. Mice *Ascl1* were amplified with Tet-O-FUW-Ascl1 mutant template and the following pairs of PCR primers: *ASCL1*-fwd: ATAGCTGCTAGCATGGAGAGCTCTGGCAAGAT and *ASCL1*-rev: TTACTTGGCGCGCCGAACCAGTTGGTAAAGTCCA; *EGFP* were amplified with pBI-EGFP-MnSOD (Addgene, 16612) and the following pairs of PCR primers:*EGFP*-fwd: ATAGCTGCTAGCATGGTGAGCAAGGGCGA and *EGFP*-rev: TTACTTGGCGCGCCCTTGTACAGCTCGTCCATGC; After being confirmed by sanger DNA sequencing, the obtained DNA fragments were ligated into the AscI and NheI restriction enzyme sites of AAV-pEF1α-FLEX-WPRE-pA plasmid. To construct a seamless ligation of *ASCL1* and *EGFP*, the PCR products from above *ASCL1* was digested with BamH1 and NheI, then was collected by gel extraction. *ASCL1* overlapped *EGFP* was amplified with pBI-EGFP-MnSOD (Addgene, 16612) and the following pairs of PCR primers: *ASCL1* overlapped_*EGFP*-fwd: TGTCGGATCCTACGACCCTCTTAGCCCAGAGGAACAAGAGCTGCTGGACTTTACCAACTGGTTCATGGTGAGCAAGGGCGA and *ASCL1* overlapped_*EGFP* -rev: TCTTACTTGG CGCGCCCTTGTACAGCTCGTCCATGC; the PCR product was digested by BamH1 and AscI, the above obtained DNA fragments were ligated into the AscI and NheI restriction enzyme sites of AAV-pEF1α-FLEX-WPRE-pA plasmid. The listed AAV vectors were packaged at the Boston Children’s Hospital Viral Core. In addition, the following were used (AAV coat serotypes, titer viral molecules/ml): AAV-FLEX-ASCL1-GFP (DJ, 2.2 × 10^14^), AAV-FLEX-GFP (DJ, 1.9 × 10^14^). Viral aliquots were stored at −80 °C before stereotaxic injection.

### Stereotaxic injection

Twelve-week-old mice were anaesthetized with isoflurane (1.5%) in 30%/70% oxygen/nitrous oxide and fixed on a stereotaxic apparatus (KOPF model 922) with ear bars. After exposing the skull via a small incision, a small hole was drilled for injection based on coordinates to bregma. For in vivo virus or microvesicles delivery, microinjection was performed using the following stereotaxic coordinates for the lateral ventricle (AAVs for left lateral ventricle, microvesicles for right lateral ventricle): anteroposterior: 0.5 mm from bregma; lateral: 0.8 mm from bregma; depth: 2.5 mm from the skull surface. Viruses were delivered via a 5 μl microliter syringe (Hamilton, 80330) over a period of 10 min; the syringe was not removed until 15–20 min after the end of infusion, to allow diffusion of the viruses. All AAVs, MVs and liposome were injected at a total volume of ~3 μl. Following stereotaxic injection, mice were individually housed with ad libitum access to regular chow diet and water.

### Foot-fault test

We used the grid containing approximately 32 cm × 20 cm × 50 cm (length × width × height) with 11- × 11-mm diameter openings^62^. Behavior was video-taped for 2–5 min to walk atop the elevated wire surface. A step was considered a foot fault if it was not providing support and the foot went through the grid hole. We started with *n* = 12 mice per group and by the end of the 14-day period, we ended up with 8 AAV-FLEx-GFP controls and 9 AAV-FLEx-ASCL1-GFP mice.

### Adhesive removal test

Mice were placed in the testing box, for a habituation period of 100 s. Two adhesive tape strips were gently applied with equal pressure on each animal paw. Mice were replaced in the testing box and recorded the time they reacted to the presence of the adhesive tape strips and removed the adhesives by the other forelimb as described^63^.

### Statistical analysis

Results were expressed as mean ± SEM and all statistical parameters and analysis are mentioned in the figure legends respectively. All experiments were performed with randomization of group assignment via 4 number lottery draw, allocation concealment, blinding of operators, blinding of measurements. Data for all experiments were analyzed with GraphPad Prism 8.0 software. *P* values of *p* < 0.05 were considered statistically significant.

### Reporting summary

Further information on research design is available in the Nature Portfolio Reporting Summary linked to this article.

## Supplementary information

Supplementary Information

Description of Additional Supplementary Files

Supplementary Movie 1

Supplementary Movie 2

Supplementary Movie 3

Supplementary Movie 4

Reporting Summary

## Data Availability

All data in this study are available in the manuscript and the Supplementary materials. Source data are provided with this paper.

## Source data

Source Data
