# Supplementary material for: Endothelial cells regulate astrocyte to neural progenitor cell trans-differentiation in a mouse model of stroke
Source: Nat Commun. 2022 Dec 19;13:7812. doi: 10.1038/s41467-022-35498-6 (PMC9763251; doi:10.1038/s41467-022-35498-6)
Supplement: Supplementary file 1 — Supplementary Information [file 41467_2022_35498_MOESM1_ESM.pdf]

SUPPLEMENTARY DATA AND FIGURE LEGENDS

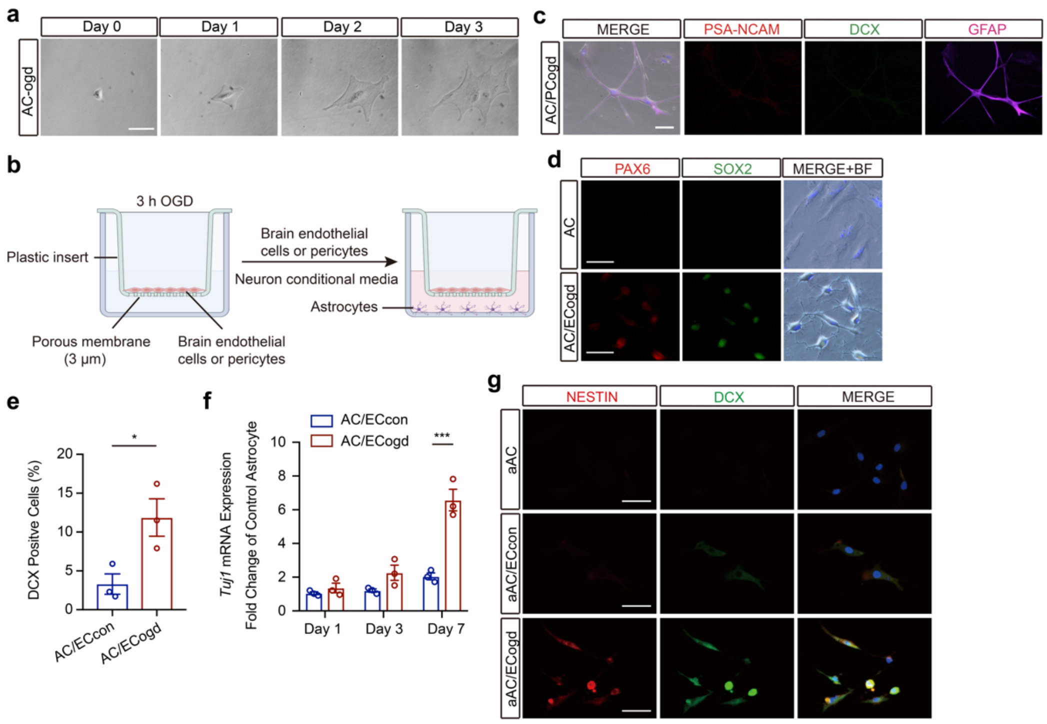

Supplementary Figure 1. Oxygen-glucose deprivation-stimulated brain endothelial cells convert astrocytes into neural progenitor cells

a Morphologic changes of same astrocytes (AC) at day 0, day 1, day 2 and day 3 post oxygen-glucose deprivation (OGD) injury. **b** Experimental schematic for either co-culturing OGD-stimulated brain endothelial cells or pericytes with astrocytes or transferring neuron conditional media onto recipient astrocytes. Created with BioRender.com. **c** Representative immunostaining of DCX, PSA-NCAM, and GFAP at day 3 post AC co-cultured with OGD-stimulated pericytes (PCogd). **d** Representative immunostaining of SOX2 and PAX6 in control AC or at day 3 post AC co-cultured with OGD-stimulated brain endothelial cells (ECogd); BF: bright filed. **e** Quantification of DCX+ cells; n=3 biologically independent samples; p=0.0365. **f** mRNA levels of *TUJ1* at day 1, day 3, and day 7 post AC co-cultured with ECcon or ECogd; n=3 biologically independent samples; p<0.0001. **g** Representative immunostaining of DCX and Nestin in control adult astrocytes (aAC) or at day 3 post aAC co-cultured with ECcon or ECogd. Experiment was repeated independently 3 times with similar results (**a**, **c-d**, **g**). Data are show mean+/-SEM. Unpaired two-tailed t-test (**e**); two-way ANOVA with post-hoc Tukey adjustment (**f**). Scale bar, 50 μm.

a

Quantification	General Characterization	Characterization of Single Vesicle	Topology of EV-Associated Components
a. Source: number of cultured cells;<br>b. EV preparation: total protein amount and total particle number;	a. Transmembrane or GPI-anchored proteins associated to plasma membrane and/or endosomes (CD63 etc.);<br>b. Cytosolic proteins recovered in EVs (Alix, TSG101 etc.);<br>c. Major components of non-EV co-isolated structures (APOA1/2 etc.);	a. Techniques providing images of single EV at high resolution (electron microscopy etc.);<br>b. Single particle analysis techniques that estimate biophysical features of EVs from other techniques than high-resolution images (nanoparticle tracking analysis etc.);	Whether component is luminal or on/at the surface of EVs.

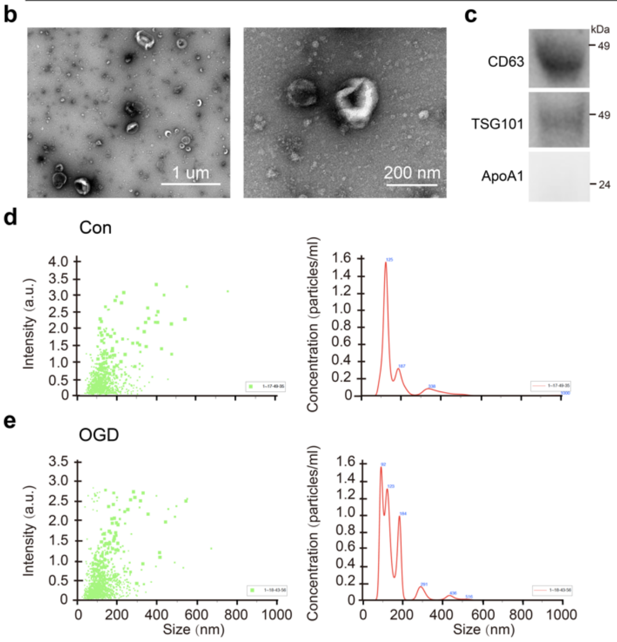

Supplementary Figure 2. Characterization of microvesicles derived from brain endothelial cells

a Characterization of microvesicle (MV) following Minimal Information for Studies of Extracellular Vesicles 2018 (MISEV2018) guidelines. **b** Electron microscopy image of MVs from brain endothelial cells. **c** Protein levels of transmembrane protein associated to plasma membrane (CD63), cytosolic protein recovered in extracellular vesicles (TSG101), and lipoprotein which is a major component of non-extracellular vesicles co-isolated structures (ApoA1) in MVs derived from brain endothelial cells. **d-e** Size distribution and concentration of MVs derived from normal (**d**) or OGD-stimulated (**e**) brain endothelial cells were detected by NanoSight. Experiment was repeated independently 3 times with similar results (**b-c**).

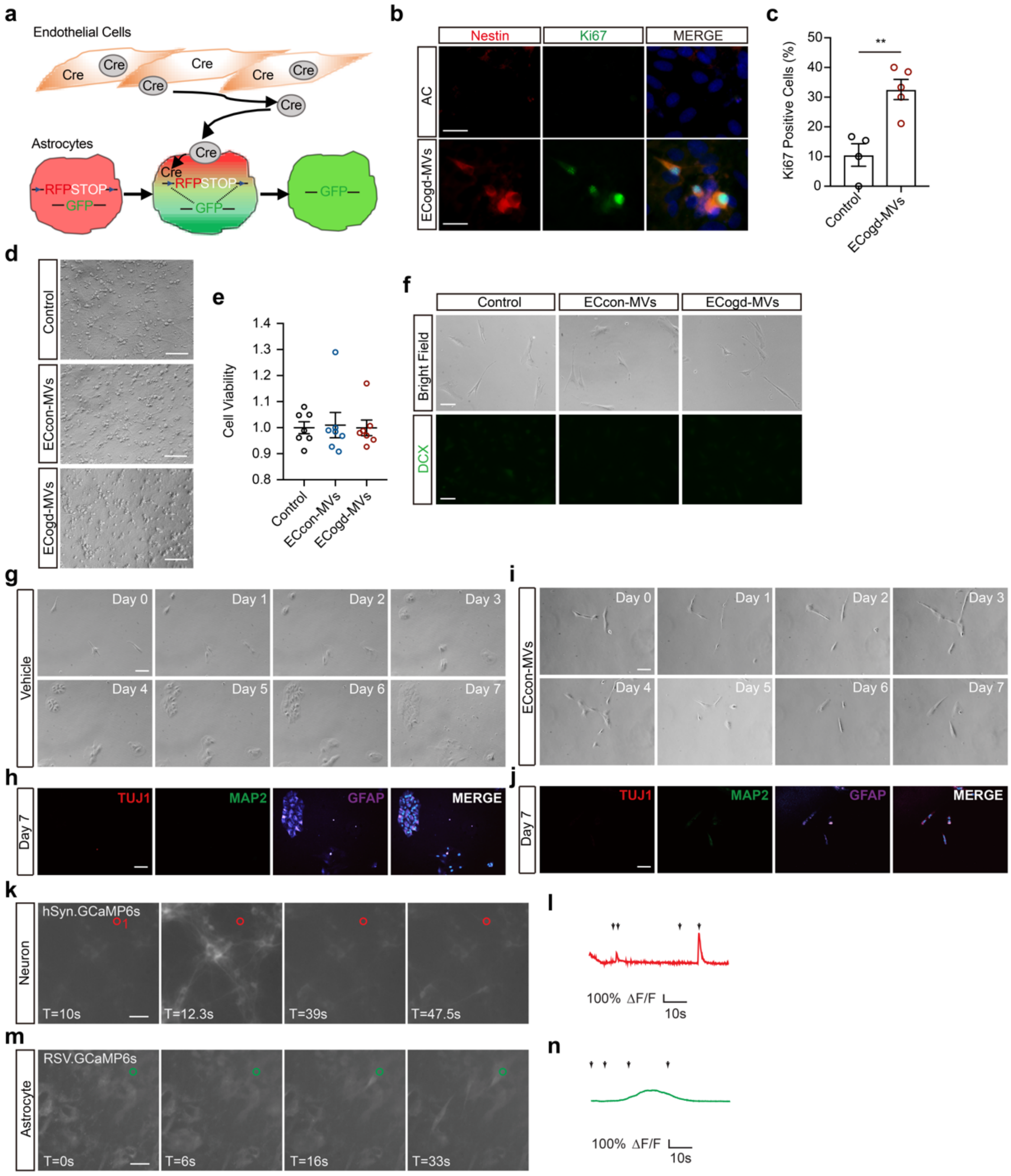

Supplementary Figure 3. Microvesicles derived from OGD-stimulated brain endothelial cells reprogram astrocytes into neural progenitor cells

a Cartoon showing the Cre-Loxp system used to report the MVs transfer of Cre recombinase (Cre) activity. A red-to-green color switch was induced in reporter+ cells (Astrocytes) upon the transfer of Cre activity derived from Cre+ cells (Endothelial cells). **b** Representative immunostaining of Nestin and Ki67 in control AC or AC treated with MVs derived from OGD-stimulated brain endothelial cells (ECogd-MVs). **c** Quantification of Ki67+ cells. n=4 biologically independent samples; p=0.0034. **d** No significant morphologic changes detected in primary neuron cultures treated with MVs derived from normal brain endothelial cells (ECcon-MVs) or ECogd-MVs. **e** Cell viability of

primary neuron cultures treated with ECcon-MVs or ECogd-MVs; n=7 samples obtained from three independent experiments. **f** DCX signal could not be detected in pericytes treated with ECcon-MVs or ECogd-MVs. **g** Morphologic changes of same AC treated with vehicle at different time points. **h** Cells in (**g**) were fixed at day 7 post vehicle treatment; Tuj1, Map2 and GFAP were detected by immunostaining. **i** Morphologic changes of same AC treated with ECcon-MVs at different time points. **j** Cells in (**i**) were fixed at day 7 post ECcon-MVs treatment; Tuj1, Map2 and GFAP were detected by immunostaining. **k** Primary neurons transduced with hSyn.GCaMP6S AAV. **l** GCaMP6s traces for region of interest; Arrows indicate the timepoints in (**k**). **m** Astrocytes transduced with RSV.GCaMP6s lentivirus. **n** GCaMP6s traces for region of interest; Arrows indicate the timepoints in (**m**). Experiment was repeated independently 3 times (**f-j**, **m-n**) or 2 times (**k-l**) with similar results. Data are shown as mean+/-SEM. Unpaired two-tailed t-test (**c**). Scale bar, 25 μm (**b**); 200 μm (**d**); 50 μm (**f-j**, **k**, **m**).

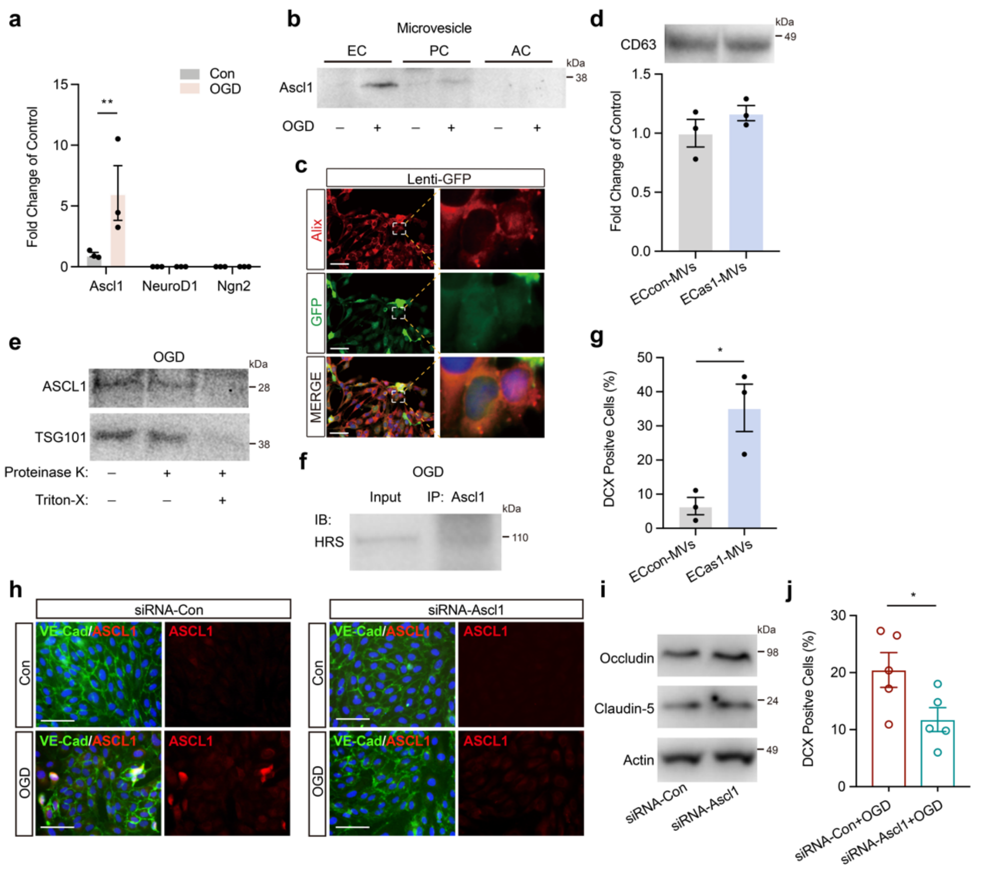

Supplementary Figure 4. Brain endothelial cells-derived microvesicles containing pro-neural transcription factor Ascl1 initiate astrocytes trans-differentiation

a mRNA levels of *Ascl1*, *NeuroD1*, and *Ngn2* in normal brain endothelial cells or OGD-stimulated brain endothelial cells. n=3 biologically independent samples. p=0.0065. **b** Protein levels of Ascl1 in MVs derived from brain endothelial cells (EC), pericytes (PC), and astrocytes (AC). **c** Representative immunostaining of Alix and GFP in brain endothelial cells transduced with control GFP lentivirus. **d** Protein levels of CD63 in microvesicles from similar cell number of normal brain endothelial cells (ECcon-MVs) or Ascl1 overexpressed brain endothelial cells (ECas1-MVs); n=3 biologically independent samples. **e** Protein levels of Ascl1 and TSG101 in microvesicels from brain endothelial cells under different treatments (100 ng/ml protease K or 100 ng/ml protease K plus 1% Triton-X incubating at 37 °C for 30 mins) indicated that Ascl1 was luminal. **f** Co-Immunoprecipitation indicated that Ascl1 could bind with endosomal sorting complex required for transport-0 (ESCRT-0) subcomplex component HRS after OGD injury (Note: our study is focused on the primary phenomenon and translational potential of brain endothelial cells providing non-cell autonomous signals for astrocyte trans-differentiation into neural progenitors after stroke. We acknowledge that we do not focus on the basic mechanisms of endothelial microvesicle loading and processing per se. Fully dissecting these microvesicle mechanisms should be warranted in future investigations.). **g** Quantification of DCX+ cells; n=3 biologically independent samples; p=0.0176. **h** Representative immunostaining of Ascl1 and VE-cadherin in brain endothelial cells transfected with control siRNA or Ascl1 siRNA. **i** Western blot analysis of tight junctions-occludin and claudin-5 in brain endothelial cells transfected with control siRNA or Ascl1 siRNA. **j** *In vitro* “loss-of-function”; n=5 samples obtained from three independent experiments; p=0.0463; The percentage of DCX+ cells in AC treated with MVs derived from OGD-stimulated but Ascl1-silenced brain endothelial cells was significantly decreased when compared to OGD-plus-control siRNA cells (Note: Since there will be multiple pathways that underlie neurogenesis after stroke, an *in vivo* “loss-of-function” experiment may not be conclusive. We acknowledge that endothelial regulation is not *required* for astrocyte trans-differentiation after stroke, but rather endothelial regulation *contributes* to astrocyte trans-differentiation after stroke. The potential translational importance for this phenomenon may be supported by our 4 “gain-of-function” experiments: infusion of endothelial microvesicles into wild type or crossed tamoxifen-inducible *Aldh1l1CreER*^{T2}*;R26R-YFP* reporter mice; and endothelial overexpression of *Ascl1* in *Tie2-cre* mice or *VE-cadherin-Cre* mice.). Experiment was repeated independently 3 times with similar results (**b-c**, **e-f**, **h-i**). Data are shown as

mean+/-SEM. Two-way ANOVA with post-hoc Bonferroni adjustment (**a**); unpaired two-tailed t-test (**g**, **j**). Scale bar, 50 μm.

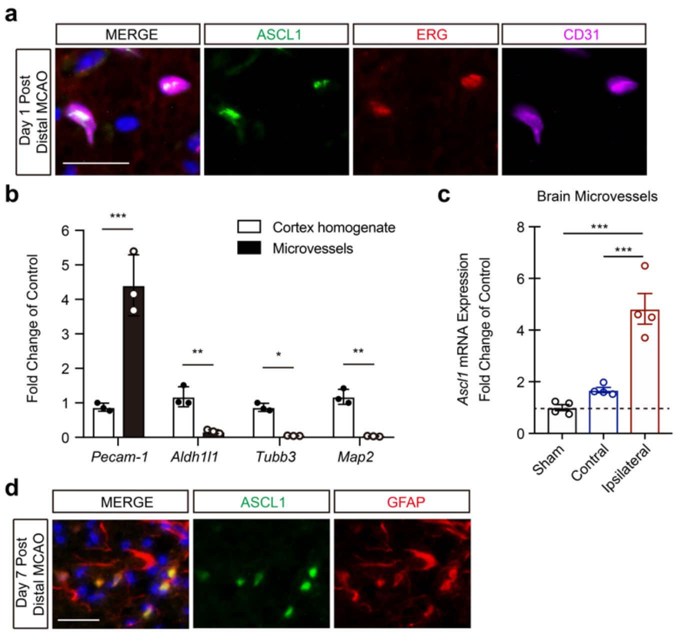

Supplementary Figure 5. Pro-neural transcription factor Ascl1 is expressed in brain microvessels after focal cerebral ischemia in mice

a Representative immunostaining of Ascl1, ERG and CD31 at day 1 post permanent distal middle cerebral artery occlusion (MCAO). **b** mRNA levels of endothelial cell marker (*Pecam-1*), astrocyte marker (*Aldh1l1)* and neuron markers *(Tubb3*, *Map2)* in cortex homogenate and microvessels; n=3 mice per group; p<0.0001 for *Pecam-1;* p=0.0094 for *Aldh1l1;* p=0.037 for *Tubb3;* p=0.0034 for *Map2.* **c** mRNA levels of *Ascl1* in brain microvessels isolated from peri-infarct area at 2 h post 60 mins transient MCAO; n=4 mice per group; Sham *vs* Ipsilateral group: p<0.0001; Contral *vs* Ipsilateral: p=0.0004. **d** Representative immunostaining of Ascl1 and GFAP at day 7 post permanent distal MCAO. Experiment was repeated independently 3 times with similar results (**a**, **d**). Data are shown as mean+/-SEM. Two-way ANOVA with post-hoc Bonferroni adjustment (**b**); one-way ANOVA with post-hoc Tukey adjustment (**c**). Scale bar, 25 μm.

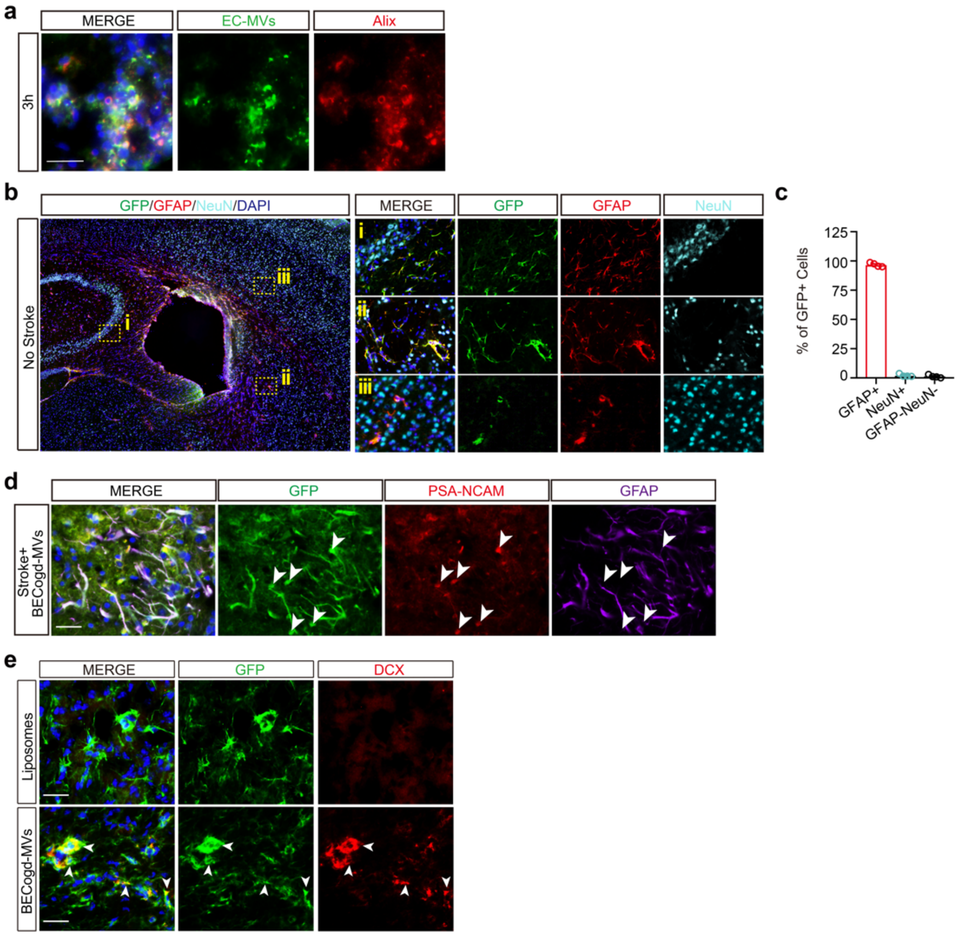

Supplementary Figure 6. Microvesicles derived from OGD-stimulated brain endothelial cells convert astrocytes into neural progenitor cells in mouse focal cerebral ischemia

a Representative immunostaining of labeled brain endothelial cells-derived microvesicles (EC-MVs) and Alix. **b** Representative immunostaining of GFP, GFAP and NeuN at day 14 post lateral ventricle injection of AAV.GFA104.PI.eGFP. **c** Quantification of GFP+ cells (totally 3 ROIs per mouse, 4 mice per group). **d** Representative immunostaining of GFP, PSA-NCAM and GFAP in striatum at day 10 post 45 mins transient MCAO. **e** Representative immunostaining of GFP and DCX in striatum at day 10 post 45 mins transient MCAO. Experiment was repeated independently 3 times (**a**, **d**) or 4 times (**e**) with similar results. Data are shown as mean+/-SEM. Scale bar, 25 μm (**a**, **d**, **e**); 200 μm (**b**).

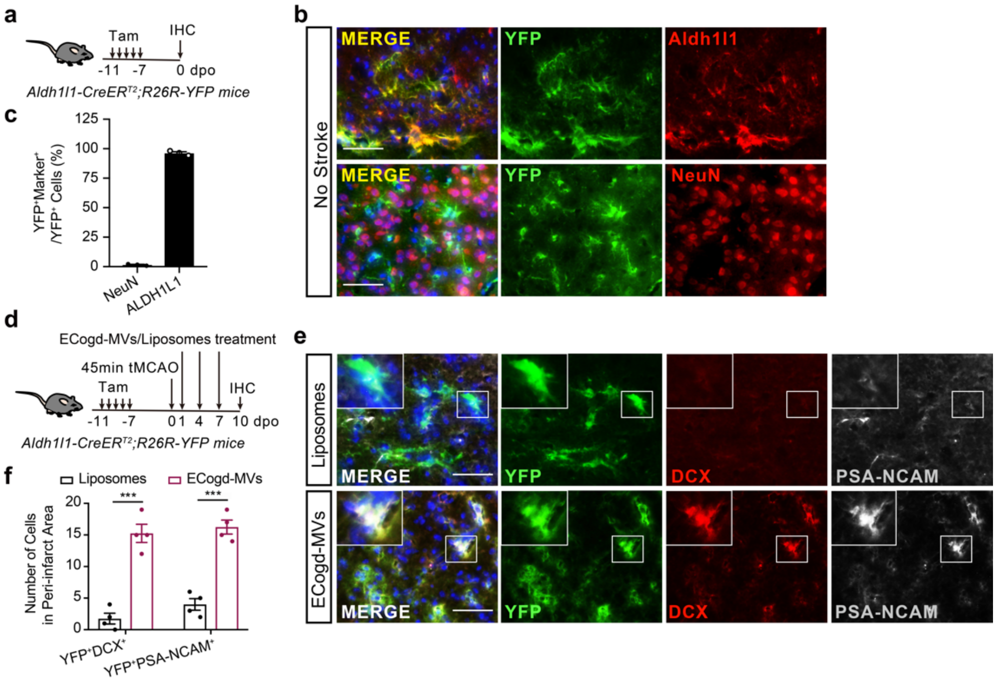

Supplementary Figure 7. Microvesicles derived from OGD-stimulated brain endothelial cells convert genetically traced astrocytes into neural progenitor cells in mouse focal cerebral ischemia

a Experimental design to trace resident astrocytes with the YFP reporter. Tam, tamoxifen; dpo, days post occlusion (n=3 mice were used). **b** Representative immunostaining of YFP, ALDHL1L, and NeuN. **c** Quantifications showing astrocyte-restricted YFP expression (totally 3 sections per mouse, 3 mice per group). **d** Experimental design to trace resident astrocytes with the YFP reporter after focal cerebral ischemia (n=4 mice were used per group). **e** Representative immunostaining of YFP, DCX, and PSA-NCAM. **f** Quantifications of YFP+ and DCX+ cells, YFP+ and PSA-NCAM+ cells in the peri-infarcted area (totally 3 sections per mouse, 4 mice per group); $p<0.0001$. Data are shown as mean+/-SEM. Two-way ANOVA with post-hoc Tukey adjustment. Scale bar, 50 µm.

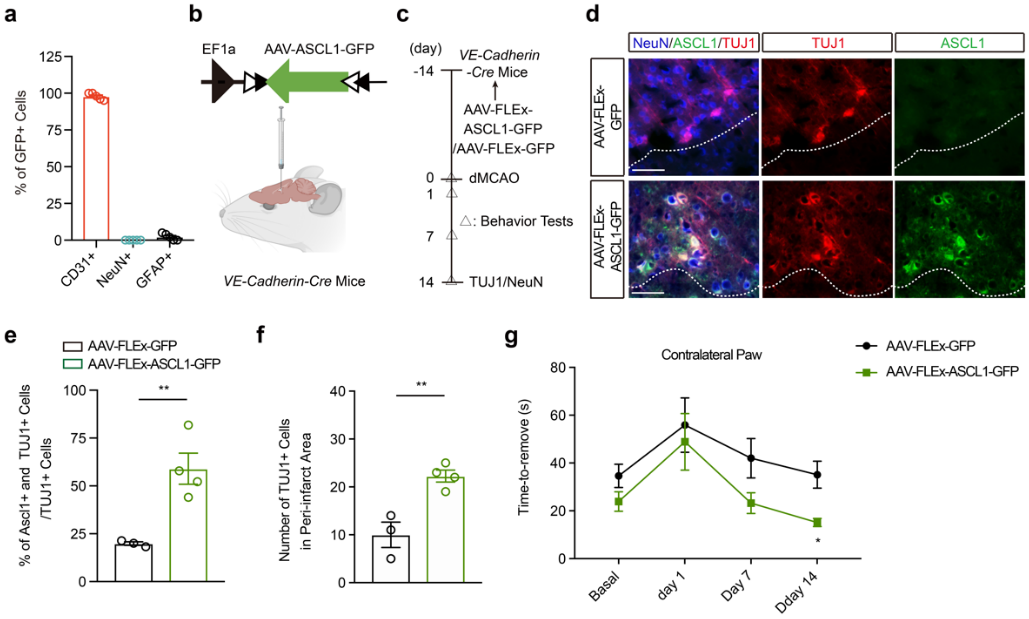

Supplementary Figure 8. Overexpression of *Ascl1* in brain endothelial cells increases neurogenesis and improves neurological recovery after focal cerebral ischemia: replication using *VE-Cadherin-Cre* mice

a Quantification of GFP+ cells (totally 3 ROIs per mouse, 5 mice per group). **b** AAV-FLEx-ASCL1-GFP or AAV-FLEx-GFP was injected into lateral ventricle of the *VE-Cadherin-Cre* mice. Created with BioRender.com. **c** Experimental design (n=8 mice were used per group). **d** Representative immunostaining of Ascl1, TUJ1, and NeuN at day 14 post permanent distal MCAO. **e** Quantification of TUJ1+ cells expressing *Ascl1* in the peri-infarct area (totally 3 sections per mouse, n=3 mice for AAV-FLEx-GFP group, n=4 mice for AAV-FLEx-ASCL1-GFP group); p=0.0098. **f** Quantification of TUJ1+ cells in the peri-infarct area (totally 3 sections per mouse, n=3 mice for AAV-FLEx-GFP group, n=4 mice for AAV-FLEx-ASCL1-GFP group); p=0.0058. **g** Tape removal test; n=8 mice per group (AAV-FLEx-ASCL1-GFP treatment induced a significant decrease of time-to-remove in contralateral paw at day 14 when compared to day 1. No significant difference was detected in AAV-FLEx-GFP group; p=0.0381).

Experiment was repeated independently 4 times with similar results (**d**). Data are shown as mean+/-SEM. Unpaired two-tailed t-test (**e**, **f**); two-way ANOVA with post-hoc Tukey adjustment (**g**). Scale bar, 50 μm.
