# Supplementary material for: Endothelial cells regulate astrocyte to neural progenitor cell trans-differentiation in a mouse model of stroke
Source: Nat Commun. 2022 Dec 19;13:7812. doi: 10.1038/s41467-022-35498-6 (PMC9763251; doi:10.1038/s41467-022-35498-6)
Supplement: Supplementary file 2 — Description of Additional Supplementary Files [file 41467_2022_35498_MOESM2_ESM.pdf]

Description of Additional Supplementary Files

Supplementary Movie 1: Calcium activity in the trans-differentiated astrocytes transduced with an adeno-associated virus (AAV) that expressed GCaMP6s under the control of the neuronal synaptophysin promoter hSyn (hSyn.GCaMP6s).

Supplementary Movie 2: Calcium activity in the primary neurons transduced with an AAV that expressed hSyn.GCaMP6s.

Supplementary Movie 3: Calcium activity in the control astrocytes transduced with an AAV that expressed hSyn.GCaMP6s.

Supplementary Movie 4: Calcium activity in the control astrocytes transduced with a lentivirus expressed GCaMP6s under the ubiquitous promoter (pHAGE-RSV-GCaMP6s).
