# Supplementary material for: Endothelial cells regulate astrocyte to neural progenitor cell trans-differentiation in a mouse model of stroke
Source: Nat Commun. 2022 Dec 19;13:7812. doi: 10.1038/s41467-022-35498-6 (PMC9763251; doi:10.1038/s41467-022-35498-6)
Supplement: Supplementary file 7 — Reporting Summary [file 41467_2022_35498_MOESM7_ESM.pdf]

nature portfolio

Corresponding author(s): Eng H. Lo

Last updated by author(s): Nov 17, 2022

Reporting Summary

Nature Portfolio wishes to improve the reproducibility of the work that we publish. This form provides structure for consistency and transparency in reporting. For further information on Nature Portfolio policies, see our Editorial Policies and the Editorial Policy Checklist.

Statistics

For all statistical analyses, confirm that the following items are present in the figure legend, table legend, main text, or Methods section.

n/a	Confirmed	
☐	☒	The exact sample size (*n*) for each experimental group/condition, given as a discrete number and unit of measurement
☐	☒	A statement on whether measurements were taken from distinct samples or whether the same sample was measured repeatedly
☐	☒	The statistical test(s) used AND whether they are one- or two-sided<br>*Only common tests should be described solely by name; describe more complex techniques in the Methods section.*
☐	☒	A description of all covariates tested
☐	☒	A description of any assumptions or corrections, such as tests of normality and adjustment for multiple comparisons
☐	☒	A full description of the statistical parameters including central tendency (e.g. means) or other basic estimates (e.g. regression coefficient) AND variation (e.g. standard deviation) or associated estimates of uncertainty (e.g. confidence intervals)
☐	☒	For null hypothesis testing, the test statistic (e.g. *F*, *t*, *r*) with confidence intervals, effect sizes, degrees of freedom and *P* value noted<br>*Give P values as exact values whenever suitable.*
☒	☐	For Bayesian analysis, information on the choice of priors and Markov chain Monte Carlo settings
☒	☐	For hierarchical and complex designs, identification of the appropriate level for tests and full reporting of outcomes
☐	☒	Estimates of effect sizes (e.g. Cohen's *d*, Pearson's *r*), indicating how they were calculated

Our web collection on statistics for biologists contains articles on many of the points above.

Software and code

Policy information about availability of computer code

Data collection	The data were not collected by using a specific software.
Data analysis	GraphPad Prism 6.01; ImageJ National Institutes of Health; FlowJo and Flowing Software 2; NanoSight NTA v3.1; MATLAB version 2018b;

For manuscripts utilizing custom algorithms or software that are central to the research but not yet described in published literature, software must be made available to editors and reviewers. We strongly encourage code deposition in a community repository (e.g. GitHub). See the Nature Portfolio guidelines for submitting code & software for further information.

Data

Policy information about availability of data

All manuscripts must include a data availability statement. This statement should provide the following information, where applicable:

- Accession codes, unique identifiers, or web links for publicly available datasets
- A description of any restrictions on data availability
- For clinical datasets or third party data, please ensure that the statement adheres to our policy

All data in this study are available in the manuscript and the Supplementary materials. Source data are provided with this paper.

Human research participants

Policy information about studies involving human research participants and Sex and Gender in Research.

Reporting on sex and gender	N/A
Population characteristics	N/A
Recruitment	N/A
Ethics oversight	N/A

Note that full information on the approval of the study protocol must also be provided in the manuscript.

Field-specific reporting

Please select the one below that is the best fit for your research. If you are not sure, read the appropriate sections before making your selection.

[x] Life sciences [ ] Behavioural & social sciences [ ] Ecological, evolutionary & environmental sciences

For a reference copy of the document with all sections, see nature.com/documents/nr-reporting-summary-flat.pdf

Life sciences study design

All studies must disclose on these points even when the disclosure is negative.

Sample size	Sample size was predetermined using the software available online: https://www.danielsoper.com/statcalc/calculator.aspx?id=47 The calculation was based on Cohen's d value where SD and average were estimated from our historical and preliminary data.
Data exclusions	For in vivo studies animals that did not demonstrate a significant reduction to less than 30% baseline LDF values during MCAO , or rapid restoration of the LDF signal during reperfusion were excluded.
Replication	For in vitro experiments, each experiment was repeated at least 3 times.
Randomization	The samples/animals were randomly assigned (simple randomization with computer-generated random numbers), before starting the experiments.
Blinding	All procedures and measurements were performed in a blinded and randomized fashion.

Reporting for specific materials, systems and methods

We require information from authors about some types of materials, experimental systems and methods used in many studies. Here, indicate whether each material, system or method listed is relevant to your study. If you are not sure if a list item applies to your research, read the appropriate section before selecting a response.

Materials & experimental systems

n/a	Involved in the study	
[ ]	[x]	Antibodies
[x]	[ ]	Eukaryotic cell lines
[x]	[ ]	Palaeontology and archaeology
[ ]	[x]	Animals and other organisms
[x]	[ ]	Clinical data
[x]	[ ]	Dual use research of concern

Methods

n/a	Involved in the study	
[x]	[ ]	ChIP-seq
[ ]	[x]	Flow cytometry
[x]	[ ]	MRI-based neuroimaging

Antibodies

Antibodies used	For Western Blot:<br>Anti-Ascl1 antibody (1:100, Santa Cruz Biotechnology, sc-374104), anti-CD63 antibody (1:200, Santa Cruz Biotechnology, sc-5275), anti-Alix antibody (1:1000, Cell Signaling Technology, 2171), anti-TSG101 antibody (1:200, Santa Cruz Biotechnology, sc-7964), anti-ApoA1 antibody (1:200, Santa Cruz Biotechnology, sc-376818), anti-HRS antibody (1:1000, Santa Cruz Biotechnology, sc-271455), anti-Occludin antibody (1:500, Invitrogen, 71-1500), anti-Claudin-5 antibody (1:1000, Invitrogen, 35-2500) and anti-β-actin antibody (1:2000, Sigma-Aldrich, A5441).

For Immunohistochemistry:
Anti-Ascl1 antibodies, 1:100, Thermo Fisher Scientific, 14579482; anti-CD31 antibody, 1:100, BD Biosciences, 565629; anti-NeuN antibody, 1:200, Millipore, MAB377; anti-doublecortin antibody, 1:100, Cell Signaling Technology, 4604; anti-Nestin antibody, 1:100, Abcam, ab11306; anti-GFAP antibody, 1:200, Innovative Research, 13-0300; anti-MAP2 antibody, 1:100, Abcam, ab32454; anti-TUBB3 antibody, 1:100, BioLegend, 801209; anti-GFP antibody, 1:100, Santa Cruz Biotechnology, sc-9996; anti-Alix antibody, 1:100, Cell Signaling Technology, 2171; anti-PSA-NCAM, 1:100, Thermo Fisher Scientific, 14911882; anti-SOX2 antibody, 1:200, Millipore, AB5603; anti-PAX6 antibody, 1:200, Thermo Fisher Scientific, 13B10-1A10; anti-Ki67 antibody, 1:200, Abcam, ab15580

Validation

All antibodies were commercial available and characterized by manufacturers.
Anti-Ascl1 antibody (Invitrogen, 14579482) is a mouse monoclonal IgG1 antibody (Clone 24B72D11). It has been validated to detect of Ascl1 of Mouse, Rat origin by WB, IHC(P);
Anti-GFAP antibody (Invitrogen, 13-0300) is a Rat / IgG2a, kappaantibody (Clone 2.2b10). It has been validated to detect of GFAP of Human, Mouse, Rat origin by IHC(P);
Anti-PSA-NCAM antibody (Invitrogen, 14911882) is a mouse monoclonal IgM antibody (Clone 12E3). It has been validated to detect of PSA-NCAM of Human, Mouse, Rat origin by IHC(P);
Anti-PAX6 antibody (Invitrogen, 13B10-1A10) is a mouse monoclonal IgG1 antibody (Clone 13B10-1A10). It has been validated to detect of PSA-NCAM of Human by WB, IHC(P);
Anti-DCX antibody (Cell Signaling Technology, 4604) is a Rabbit monoclonal antibody and is produced by immunizing animals with synthetic peptide corresponding to human doublecortin.. It has been validated to detect of DCX of Mouse, Rat by IF.
Anti-ASCL1 Antibody (sc-374104) is a mouse monoclonal IgG2a κ, and raised against amino acids 181-236 mapping at the C-terminus of ASCL1 of human origin. It has been validated to detect of ASCL1 of mouse, rat and human origin by WB, IF;
CD63 Antibody (sc-5275) is a mouse monoclonal IgG1 κ CD63 antibody, and raised against full length CD63 of human origin
It has been validated to detect of CD63 of mouse, rat and human origin by WB, IF, IHC(P)
Tsg 101 Antibody (sc-7964) is a mouse monoclonal IgG2a κ tsg 101 antibody, and raised against amino acids 1-138 representing full length tsg 101 (tumor susceptibility gene 101) of mouse origin. It has been validated to detect of tsg 101 of mouse, rat and human origin by WB, IF, IHC(P);
Anti-Hrs Antibody (sc-271455) is a mouse monoclonal IgG1 κ Hrs antibody, and it is specific for an epitope mapping between amino acids 121-173 within an internal region of Hrs of human origin. It has been validated to detect of Hrs of mouse, rat and human origin by WB, IF ;
Anti-Alix antibody (Cell Signaling Technology, 2171) is a monoclonal antibody and is produced by immunizing animals with full-length recombinant human Alix protein. It has been validated to detect of Alix of Human, Mouse, Rat by WB.
Anti-ApoA1 antibody (SC-69755) is a mouse monoclonal IgG1 antibody, and raised against apoA-I protein of human origin. It has been validated to detect of apoA-I of mouse, rat and human origin by WB, IP, IF;
Anti-Occludin antibody (Invitrogen, 71-1500) is a rabbit polyclonal antibody, and raised against the C-terminal 150 amino acid region of human occludin. It has been validated to detect of Occludin of rat and human origin by WB, IF, IHC(P);
Anti-claudin-5 antibody (Invitrogen, 35-2500) is a mouse monoclonal IgG1 antibody, and raised against synthetic peptide. It has been validated to detect of claudin-5 of Human, Mouse, Rat origin by WB, IHC(P);
Anti-Ki67 antibody (ab15580) is a Rabbit polyclonal antibody, and raised against synthetic peptide. It has been validated to detect of Ki67 of Human, Mouse by IHC(P);
Anti-MAP2 antibody (ab32454) is a Rabbit polyclonal antibody, and raised against Synthetic peptide within Rat MAP2 aa 1-100 (N terminal) conjugated to keyhole limpet haemocyanin. It has been validated to detect of MAP2 of Rat, Mouse by IHC(P), ICC;
Anti-NeuN antibody (MAB377) is a mouse monoclonal antibody. Anti-NeuN Antibody, clone A60 detects level of NeuN and has been published and validated for use in FC, IC, IF, IH, IH(P), IP and WB for mouse and rat.
Anti-Sox2 antibody (AB5603) is a highly specific rabbit polyclonal antibody SOX2 and has been tested for use in Immunocytochemistry, and Immunohistochemistry (Paraffin), and Western Blotting for human and mouse.
Anti-GFP Antibody (sc-9996) is a mouse monoclonal IgG2a κ GFP antibody and raised against amino acids 1-238 representing full length GFP (green fluorescent protein) of Aequorea victoria origin. GFP Antibody (B-2) is validated for detection of GFP and GFP mutant fusion proteins by WB, IF.
Anti-tubb3 antibody (Biolegend, 801209) is a Mouse IgG2a, κ antibody. It has been validated to detect of tubb3 of Human, Mouse, Rat origin by ICC, ICF;

Animals and other research organisms

Policy information about studies involving animals; ARRIVE guidelines recommended for reporting animal research, and Sex and Gender in Research

Laboratory animals

C57BL6 (Jax Stock No: 000664) mice, (B6.Cg-Tg(Tek-cre)1Ywa/J (Jax Stock No: 008863) mice, B6.FVB-Tg(Cdh5-cre)7Mlia/J (Jax Stock No: 006137) mice, Aldh1l1- CreERT2 (Jax Stock No: 029655), and R26R-YFP (Jax Stock No: 006148) were obtained from the Jackson Laboratory 34, 58, 59, 60. 12-week males of mouse lines were used for experiments. All mice were (up to 4 mice per cage) maintained in a controlled pathogen-free/germ-free environment with a temperature of 68-73°F, 12/12 h light/dark cycle, 30-70% humidity, and food (Prolab Isopro RMH3000 Irradiated, 3003219-249) and water provided ad libitum. Experiments were performed under institutionally approved protocol in accordance with the National Institute of Health's Guide for the Care and Use of

	Laboratory Animals. All animals were randomly allocated to treatment groups.
Wild animals	No wild animals were used
Reporting on sex	Since female mice must be tested across the estrous cycle and are more variable than males, male mice were used in this proof-of-concept study. Female and male mice will be included in our future studies.
Field-collected samples	No field collected samples were used
Ethics oversight	All experiments were performed under approved Institutional Animal Care and Use Committee protocols (2010N000147 and 2016N000493) in accordance with National Institutes of Health guidelines and with the United States Public Health Service's Policy on Human Care and Use of Laboratory Animals and following Animals in Research: Reporting In vivo Experiments (ARRIVE) guidelines.

Note that full information on the approval of the study protocol must also be provided in the manuscript.

Flow Cytometry

Plots

Confirm that:

- [x] The axis labels state the marker and fluorochrome used (e.g. CD4-FITC).
- [x] The axis scales are clearly visible. Include numbers along axes only for bottom left plot of group (a 'group' is an analysis of identical markers).
- [x] All plots are contour plots with outliers or pseudocolor plots.
- [x] A numerical value for number of cells or percentage (with statistics) is provided.

Methodology

Sample preparation	Brains from wild type mice were collected at 2 hours after distal MCAO. Tissues were gently minced and then digested at 37° C for 30 min with an enzyme cocktail.
Instrument	BD Fortessa
Software	FlowJo and Flowing Software 2 (https://bioscience.fi/services/cell-imaging/flowing-software/)
Cell population abundance	We sorted the brain endothelial cells and neurons based on CD31 expression and L1CAM expression. Pericytes and microglia/ macrophages were sorted based on CD11b expression and CD13 expression.
Gating strategy	FACS analysis was performed using an unstained or phenotype control for determining appropriate gates, voltages, and compensations required in multivariate flow cytometry.

- [x] Tick this box to confirm that a figure exemplifying the gating strategy is provided in the Supplementary Information.
