# Supplementary figures and images for: Endothelial cells regulate astrocyte to neural progenitor cell trans-differentiation in a mouse model of stroke
Source: Nat Commun. 2022 Dec 19;13:7812. doi: 10.1038/s41467-022-35498-6 (PMC9763251; doi:10.1038/s41467-022-35498-6)

Fig. 3b

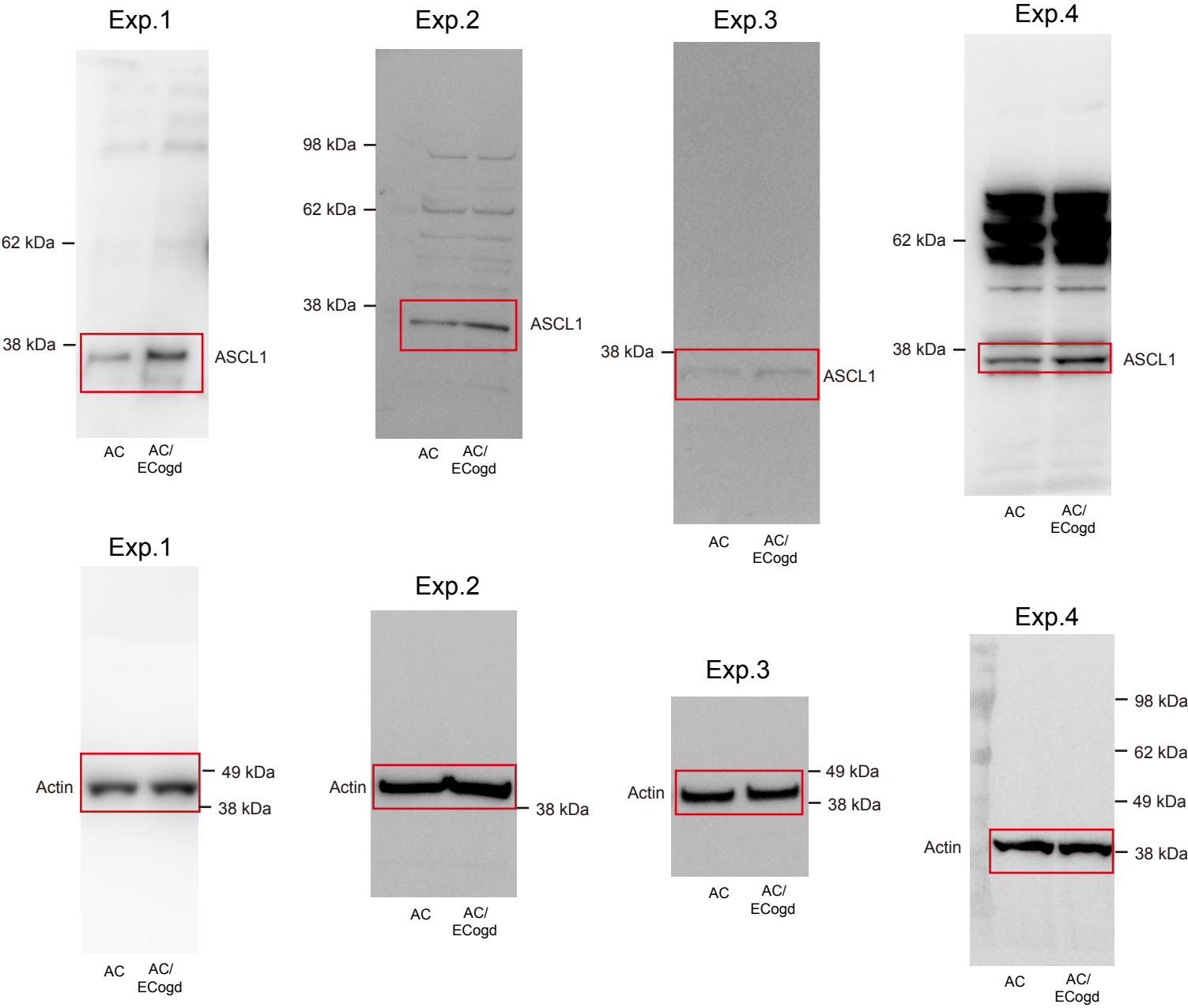

Supplement: Supplementary file 8 — Source Data [file 41467_2022_35498_MOESM8_ESM.zip › Source Data/UncroppedBlots_Fig3b.pdf]

Fig. S2c

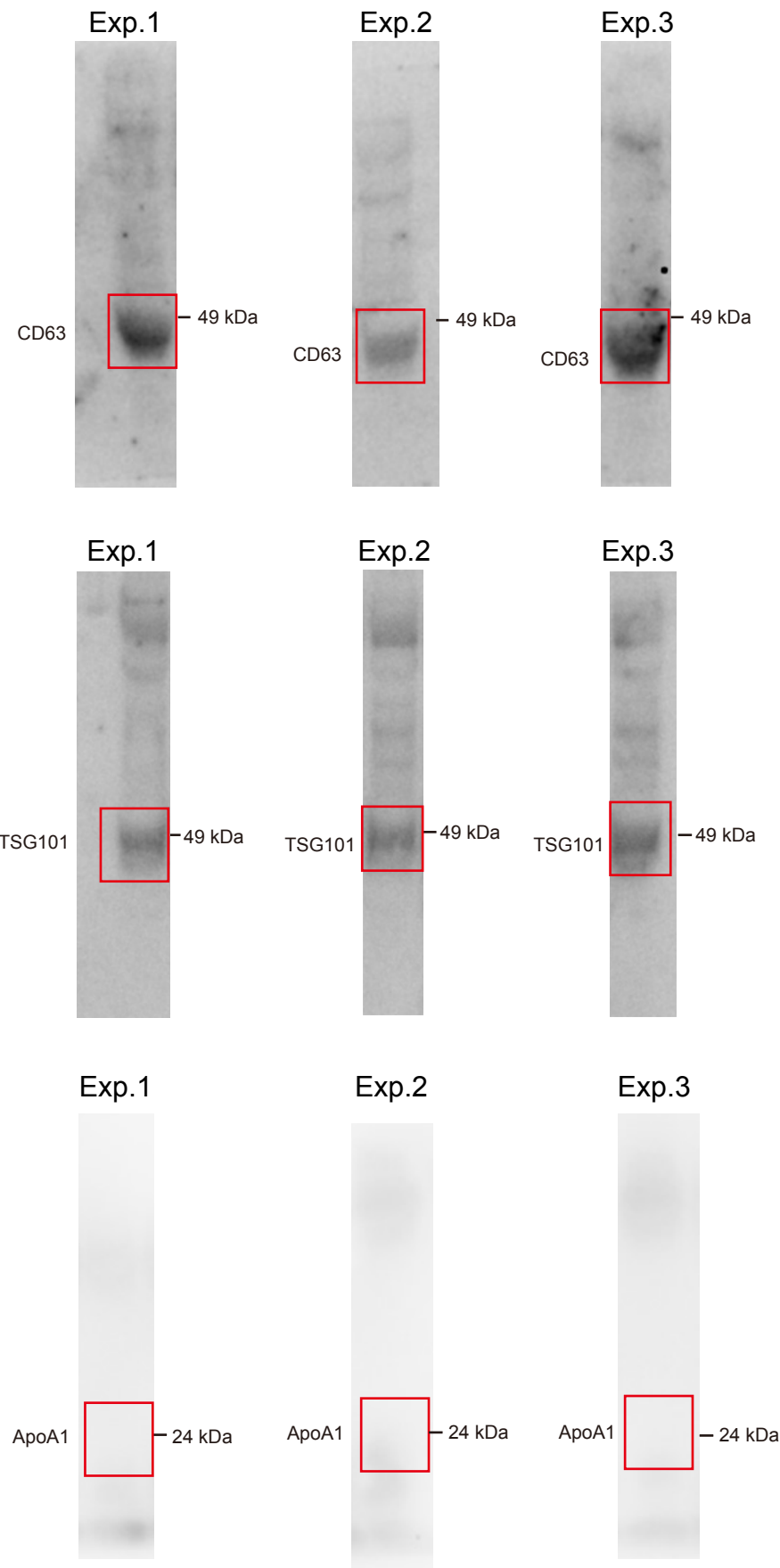

Supplement: Supplementary file 8 — Source Data [file 41467_2022_35498_MOESM8_ESM.zip › Source Data/UncroppedBlots_FigS2c.pdf]

Fig. 3e

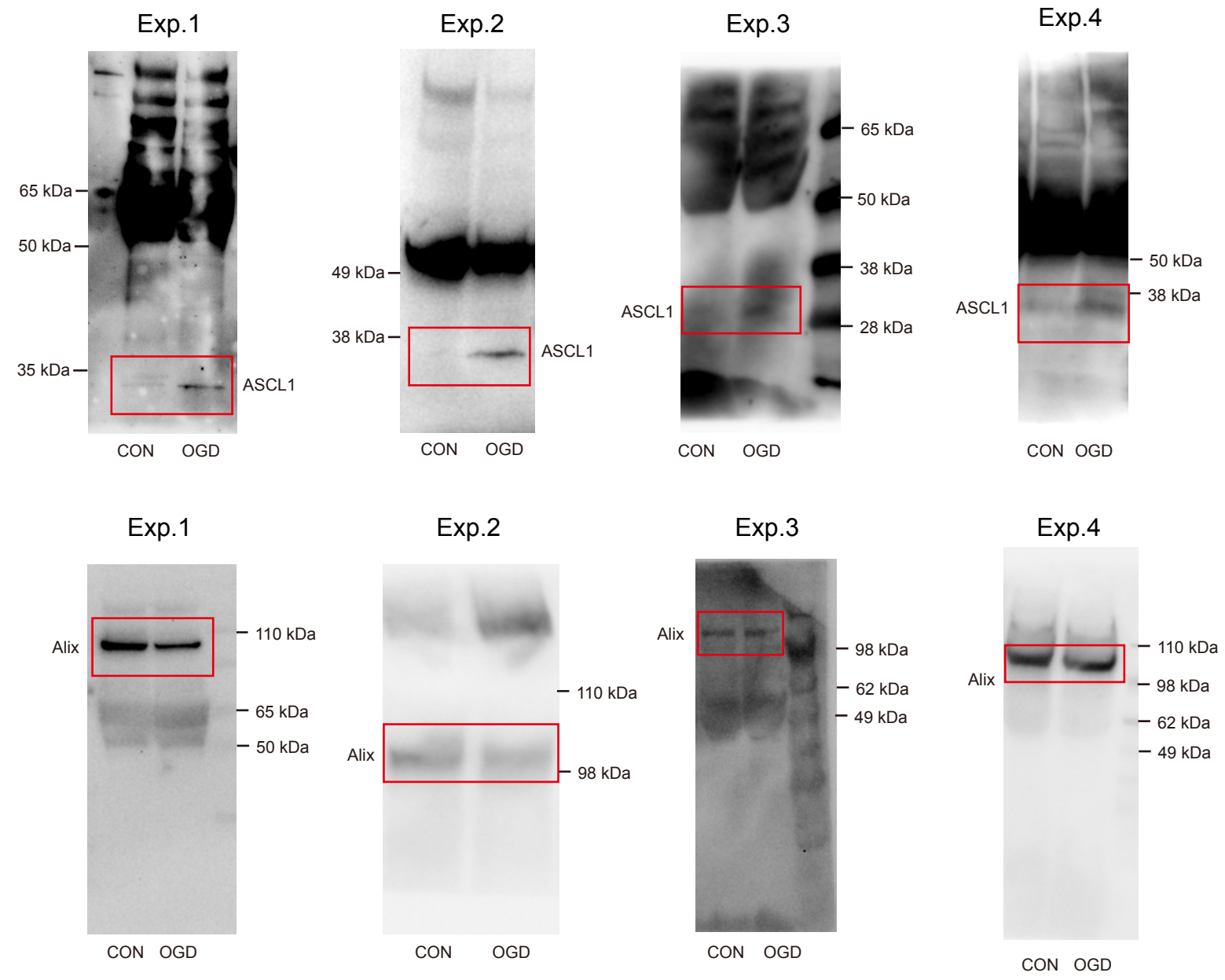

Supplement: Supplementary file 8 — Source Data [file 41467_2022_35498_MOESM8_ESM.zip › Source Data/UncroppedBlots_Fig3e.pdf]

Fig. S4b

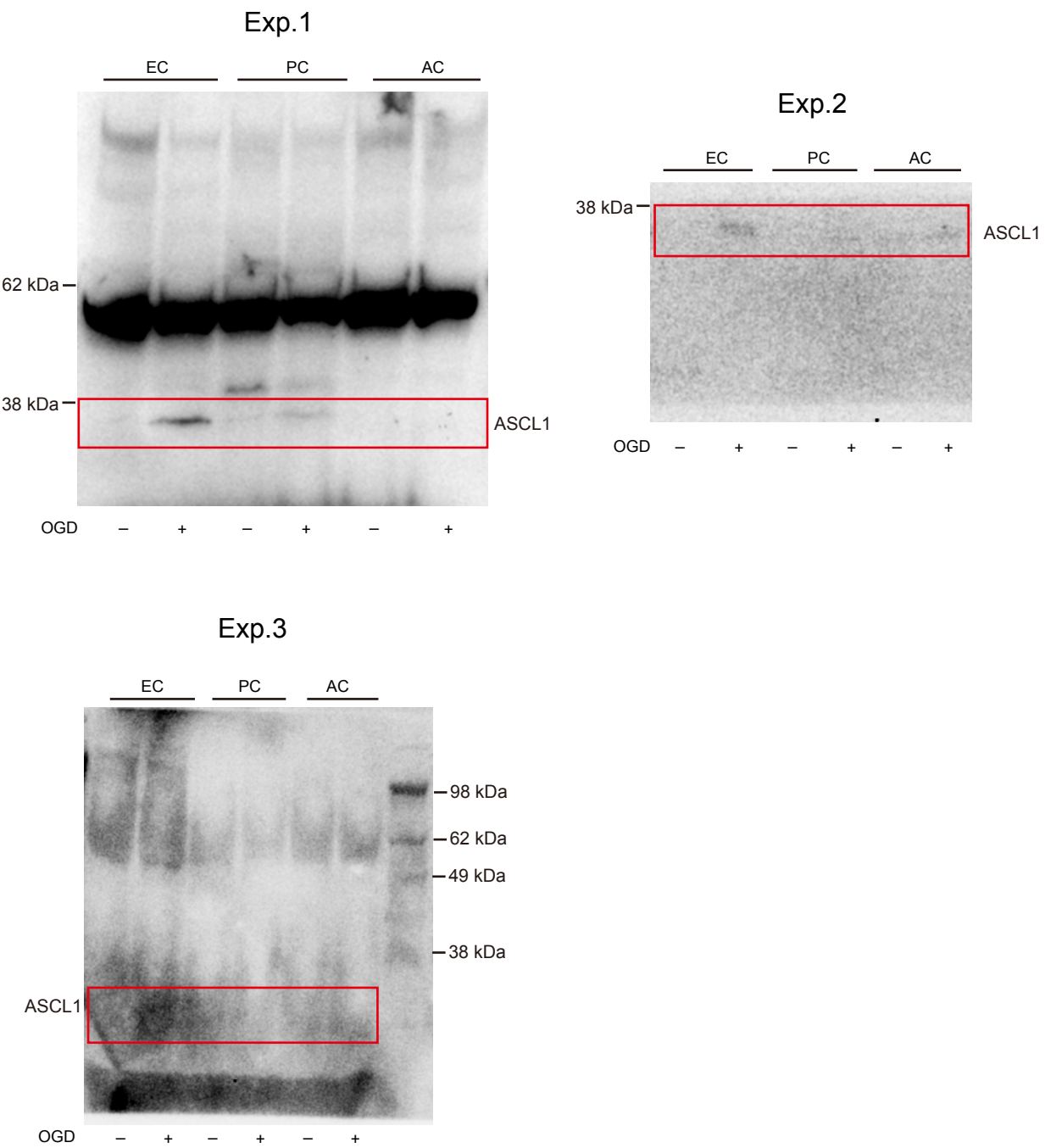

Supplement: Supplementary file 8 — Source Data [file 41467_2022_35498_MOESM8_ESM.zip › Source Data/UncroppedBlots_FigS4b.pdf]

Fig. S4d

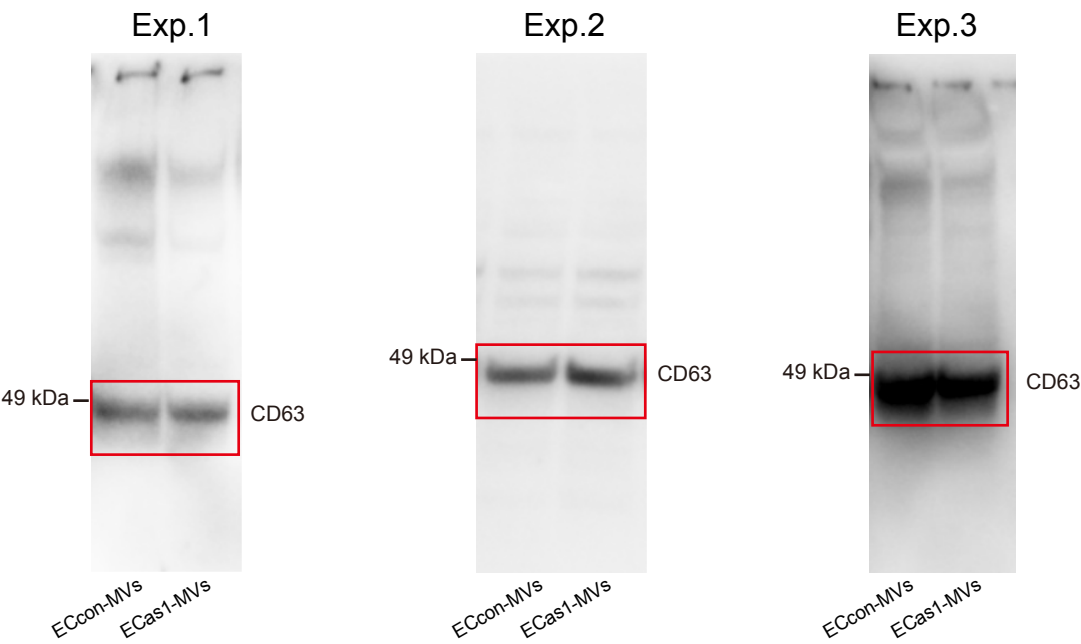

Supplement: Supplementary file 8 — Source Data [file 41467_2022_35498_MOESM8_ESM.zip › Source Data/UncroppedBlots_FigS4d.pdf]

Fig. S4e

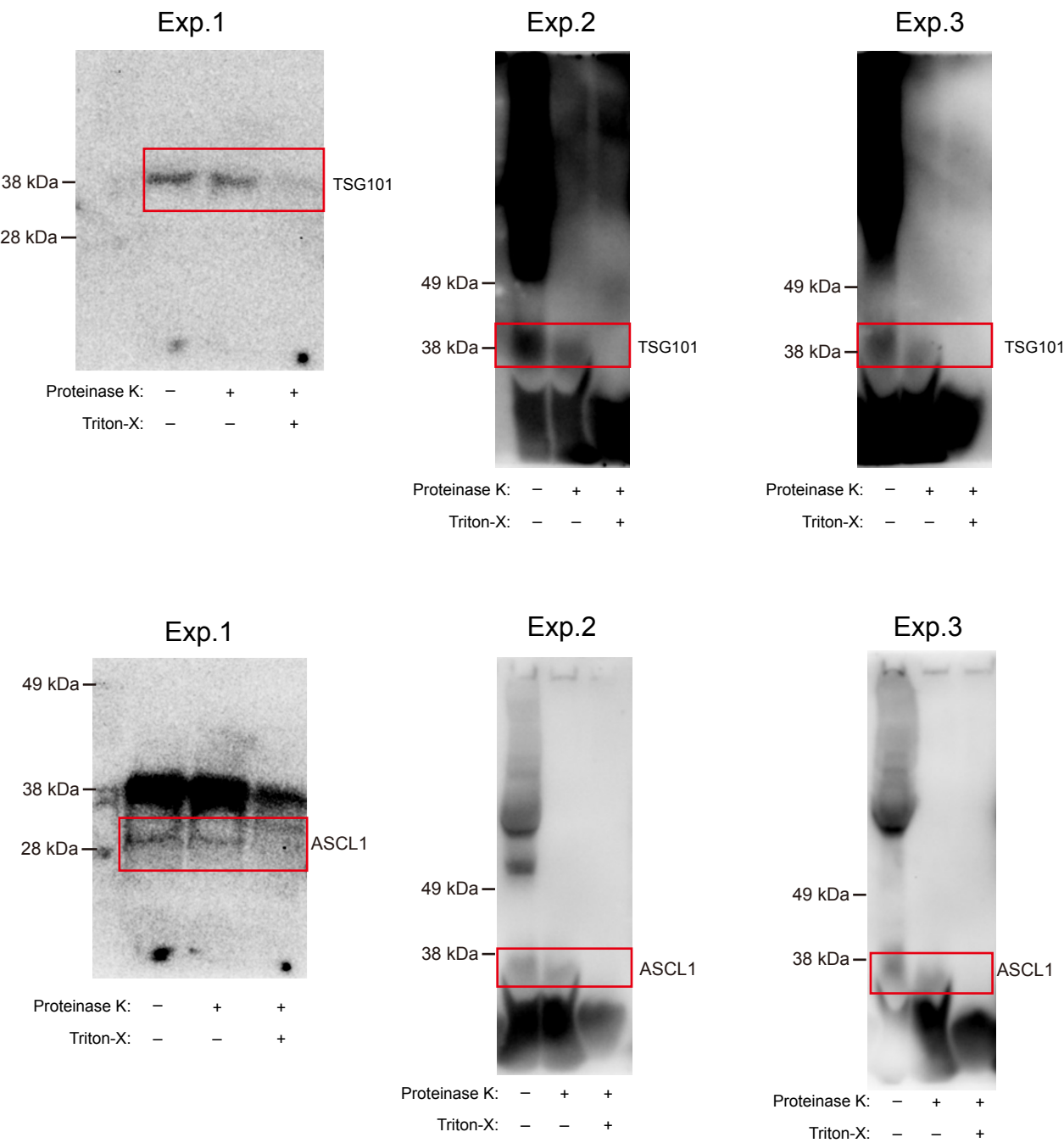

Supplement: Supplementary file 8 — Source Data [file 41467_2022_35498_MOESM8_ESM.zip › Source Data/UncroppedBlots_FigS4e.pdf]

Fig. S4f

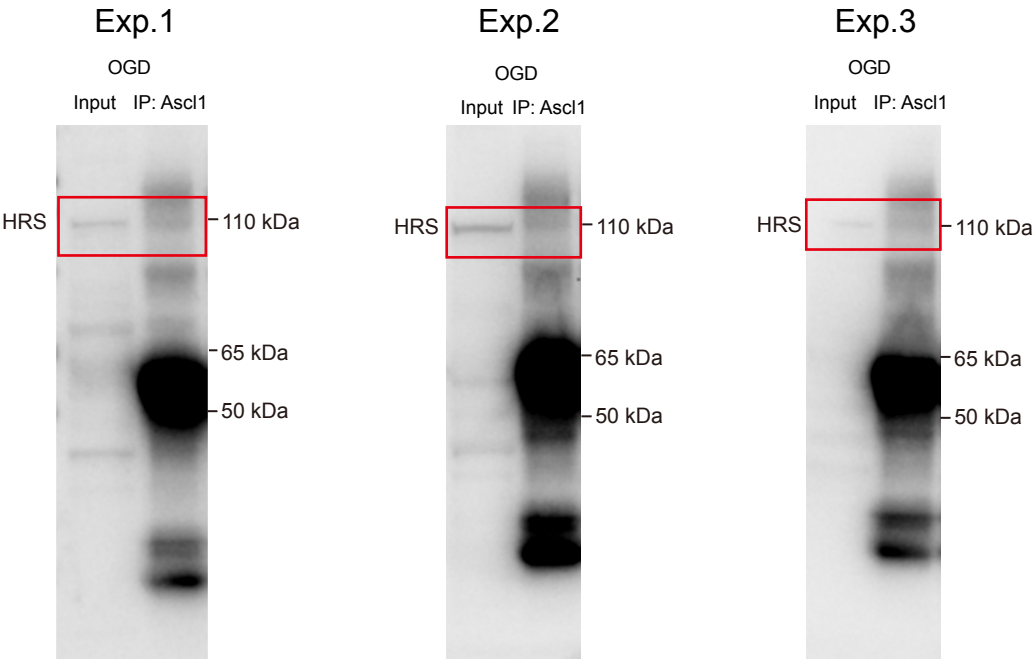

Supplement: Supplementary file 8 — Source Data [file 41467_2022_35498_MOESM8_ESM.zip › Source Data/UncroppedBlots_FigS4f.pdf]

Fig. S4i

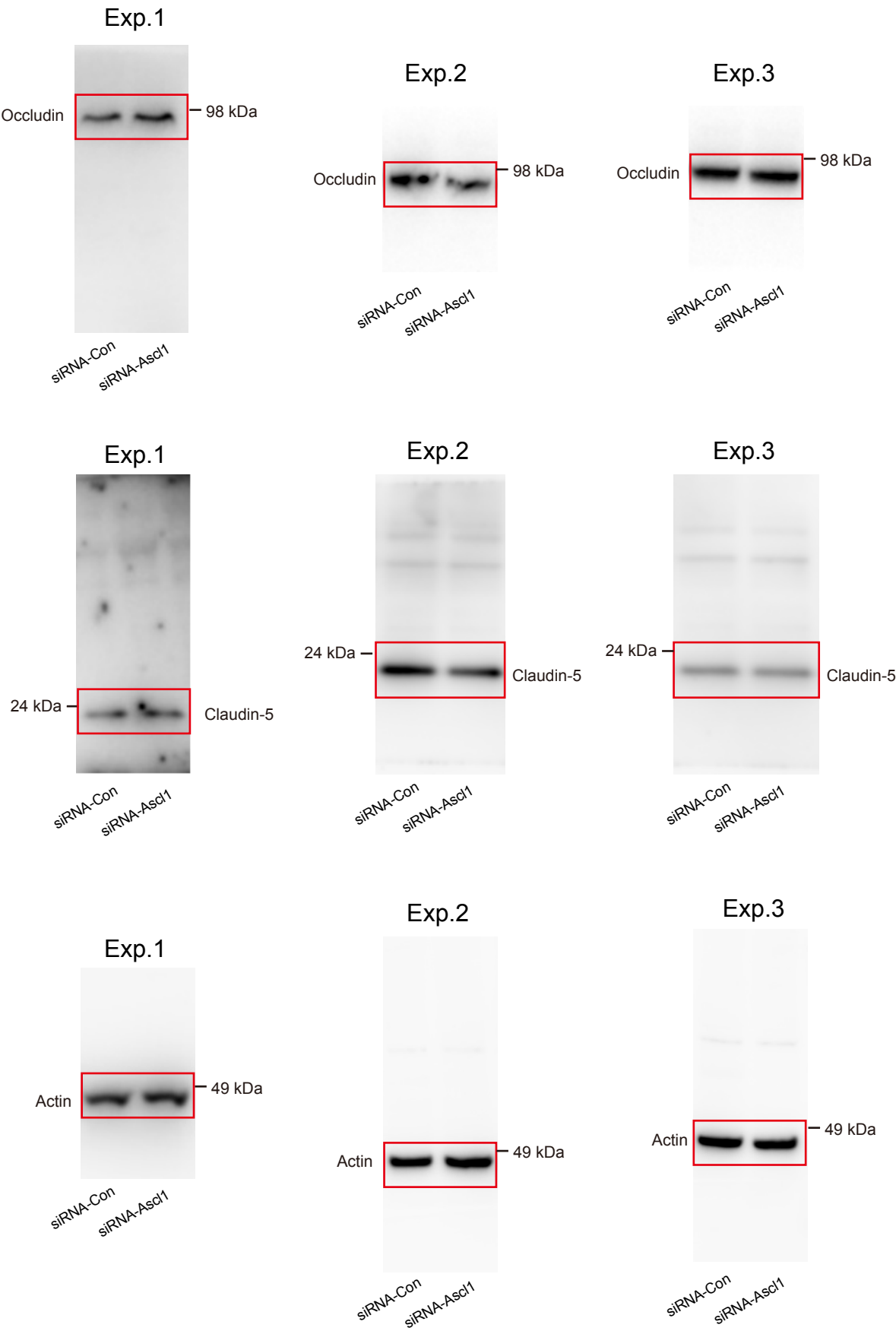

Supplement: Supplementary file 8 — Source Data [file 41467_2022_35498_MOESM8_ESM.zip › Source Data/UncroppedBlots_FigS4i.pdf]
